# The role of the motor thalamus in deep brain stimulation for essential tremor

**DOI:** 10.1016/j.neurot.2023.e00313

**Published:** 2024-01-08

**Authors:** Clemens Neudorfer, Kristy Kultas-Ilinsky, Igor Ilinsky, Steffen Paschen, Ann-Kristin Helmers, G. Rees Cosgrove, R. Mark Richardson, Andreas Horn, Günther Deuschl

**Affiliations:** a Brain Modulation Lab, Department of Neurosurgery, Massachusetts General Hospital, Boston, MA, 02114, USA; b Department of Neurosurgery, Massachusetts General Hospital, Harvard Medical School, Boston, MA, USA; c Center for Brain Circuit Therapeutics Department of Neurology Brigham & Women's Hospital, Harvard Medical School, Boston, MA, USA; d Movement Disorder and Neuromodulation Unit, Department of Neurology, Charité -Universitätsmedizin Berlin, Corporate Member of Freie Universität Berlin and Humboldt-Universität zu Berlin, Berlin, Germany; e Department of Anatomy and Cell Biology, The University of Iowa, Iowa City, IA, USA; f Department of Neurology, Christian-Albrechts-University, Kiel, Germany; g Department of Neurosurgery, Christian-Albrechts-University, Kiel, Germany; h Department of Neurosurgery, Brigham and Women's Hospital, Harvard Medical School, Boston, MA, USA

**Keywords:** Tremor, Motor thalamus, Deep brain stimulation, Human thalamic nomenclature, Parcellation, Vim

## Abstract

The advent of next-generation technology has significantly advanced the implementation and delivery of Deep Brain Stimulation (DBS) for Essential Tremor (ET), yet controversies persist regarding optimal targets and networks responsible for tremor genesis and suppression. This review consolidates key insights from anatomy, neurology, electrophysiology, and radiology to summarize the current state-of-the-art in DBS for ET. We explore the role of the thalamus in motor function and describe how differences in parcellations and nomenclature have shaped our understanding of the neuroanatomical substrates associated with optimal outcomes. Subsequently, we discuss how seminal studies have propagated the ventral intermediate nucleus (Vim)-centric view of DBS effects and shaped the ongoing debate over thalamic DBS versus stimulation in the posterior subthalamic area (PSA) in ET. We then describe probabilistic- and network-mapping studies instrumental in identifying the local and network substrates subserving tremor control, which suggest that the PSA is the optimal DBS target for tremor suppression in ET. Taken together, DBS offers promising outcomes for ET, with the PSA emerging as a better target for suppression of tremor symptoms. While advanced imaging techniques have substantially improved the identification of anatomical targets within this region, uncertainties persist regarding the distinct anatomical substrates involved in optimal tremor control. Inconsistent subdivisions and nomenclature of motor areas and other subdivisions in the thalamus further obfuscate the interpretation of stimulation results. While loss of benefit and habituation to DBS remain challenging in some patients, refined DBS techniques and closed-loop paradigms may eventually overcome these limitations.

## Introduction

The objective of this paper is to outline principal aspects of current of deep brain stimulation (DBS) targets for tremor, focusing on the motor thalamus and regions beneath it, denominated here collectively as the posterior subthalamic area (PSA).

The first part explores the historical discoveries of the motor thalamus. Groundbreaking findings from the early 20th century have shaped the current view of this important region. The ongoing debates over nomenclature reflect the continuous and clinically significant discoveries of afferent projections and the criteria essential for defining target regions from a neurobiological standpoint. New methods now enable the detailed delineation of these afferent structures to the thalamus, allowing for accurate tracing of tract systems. While histology and modern MRI-imaging represent the anatomical approach, the contribution of electrophysiology has been equally important. Indeed, its roots date back to the 1950s and have led to an electrophysiological definition of the ventral intermediate nucleus of the thalamus (Vim). The amalgamation of both, anatomical and electrophysiological knowledge has allowed us to derive our current understanding of the action of DBS on tremor.

The clinical results of DBS surgery have been critically analyzed previously with the methods of evidence-based medicine (EBM), but most of the clinical studies analyzed did not distinctly differentiate between the various targets in the Vim or PSA, with EBM analyzing the bulk of clinical studies across mixed targets. This paper updates these findings, including side effects and the habituation as possible complications.

The concluding section of this review addresses the distinction of the Vim and PSA concerning the therapeutic outcomes of DBS-interventions. Indeed, there is mounting evidence for superior short- and medium-term efficacy of PSA-over Vim-DBS. The range of imaging methods (i.e., local and network mapping approaches) used to establish this superiority of current and past studies is extensive. The latest MR-imaging methods now define the PSA for each individual and may in the long run replace targeting approaches derived from standardized anatomical atlases (e.g., the Schaltenbrand-Wahren atlas [1]). Despite this progress, there remains a pressing need for additional controlled, blinded studies – currently underway – to compare the long-term results and further substantiate these findings.

## Functional anatomy of the motor thalamus

### Discovery of thalamic involvement in motor function

**Fig. 1:**
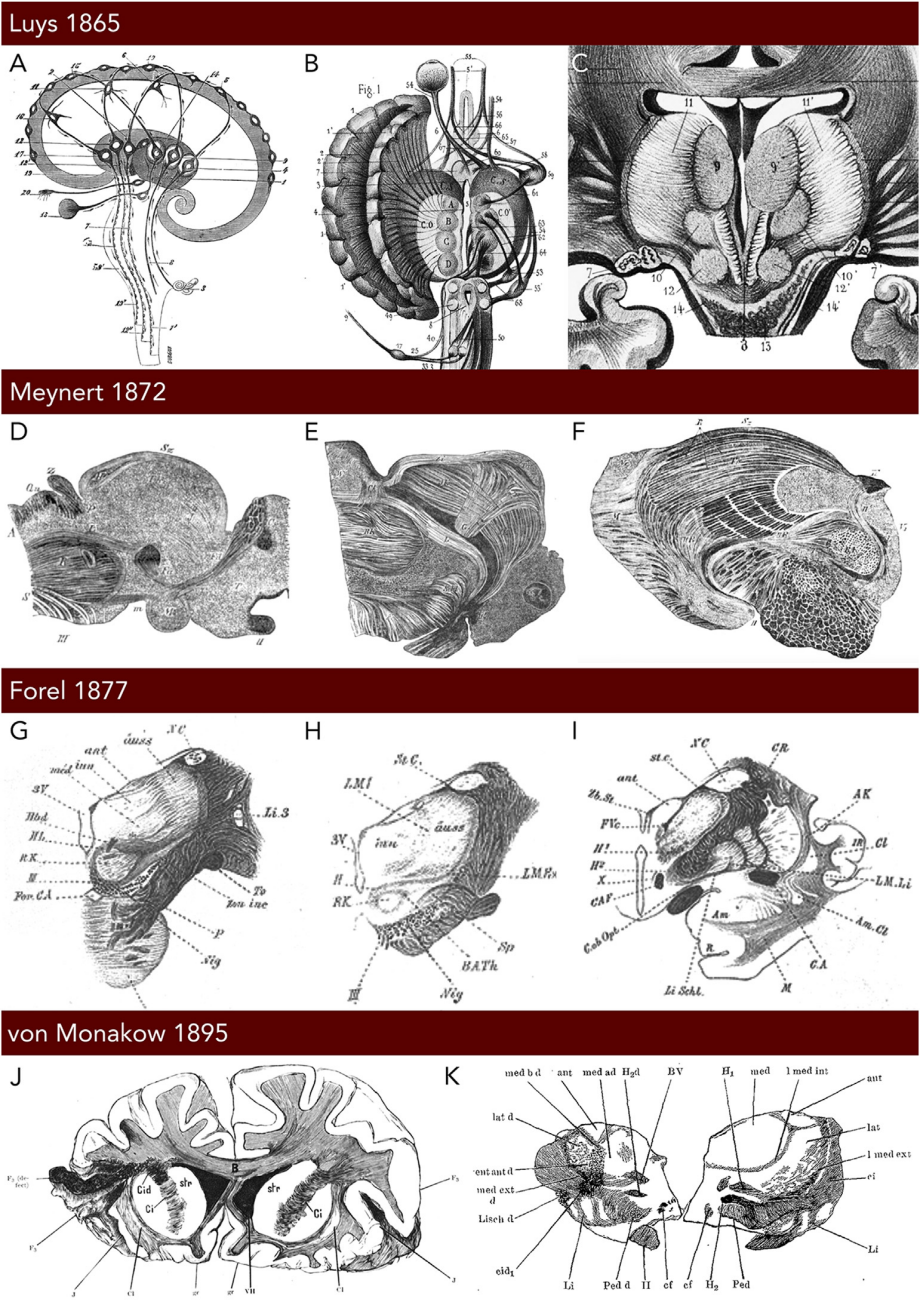
**Early depictions of the thalamus and its nuclear parcellation according. (A)** Diagram of the brain in the sagittal plane featuring the principal cellular centers (labeled 4, 9, 14, and 19) of the thalamus as hypothesized by Luys. Note the significant functional emphasis on the processing of sensory information as illustrated by the input from the olfactory bulb (20), retina (13), spinal cord (8), and the inner ear (3). Information from the thalamus is relayed to the cortex before being transmitted to the striatum (1), from where it descends to the spinal cord. Arrows represent the presumed direction of information flow. **(B)** Three-dimensional representation of the thalamus and its input and output structures, viewed in the axial plane. **(C)** Drawing of a frontal section through the human thalamus displaying the centers moyens (9, 9’) and médians (10, 10’). Sagittal **(D)**, axial **(E)**, and coronal **(F)** transparent sections by Meynert suggesting poor differentiation of individual thalamic nuclei. **(G**–**I)** Depiction of coronal transparent sections by Forel. **(J,K)** Coronal sections of a postmortem brain as reproduced by von Monakow, showing retrograde thalamic degeneration within thalamus **(K)** secondary to lesioning of the orbital and anterior insular cortex **(J)**. Adopted from Luys [3] (A–C), Meynert [14] (D–F), Forel [15] (G–I), and von Monakow [16] (J,K).

Our understanding about the role of the thalamus in motor function has been shaped by centuries of scientific exploration, experimentation, and conceptual refinement that is still ongoing today. The term ‘thalamus’, derived from the Greek for ‘inner chamber’, aptly describes the structure's position as a central integration node within the human brain. However, its involvement in motor function has only been uncovered recently. Indeed, neglecting the highly integrative role of the thalamus, early studies primarily attributed a role in sensory processing to the structure. Perhaps the first such case was reported by John Hunter in 1825 [2], who described a patient with loss of sight, hearing, and touch over a period of three years. Post-mortem examinations in this case revealed bilateral lesions encapsulating the thalamus. Luys [3], known for his work on the subthalamic nucleus, in particular, was an early proponent of the thalamus as a relay for sensory information to the cortex. Inspired by the finding of a brain ‘center’ for speech by his contemporary Paul Broca [4], he described the thalamus as the center for processing of sensory impressions. Adopting the staining methods introduced by Gerlach, Luys identified four thalamic centers which he hypothesized to serve as separate conduits for the sensory pathways (Fig. 1A–C). While Luys' delineations could not be confirmed in subsequent studies, his *centre médian* label has been adopted and remains in use in modern thalamic nomenclatures.

The development and refinement of staining methods was imperative for understanding thalamic architecture and formed the basis of the fundamental work done by Luys, Meynert (Fig. 1D–F), Forel (Fig. 1G–I), and others. Most notably, the discovery of cresyl violet by Franz Nissl in 1894 and his subsequent studies of thalamic architecture allowed him to provide a first full account of thalamic subdivisions in mammals [5]. Nissl subdivided the anterior and medial nuclei into three subnuclei, and the lateral and pulvinar nuclei into two subnuclei, respectively. He also used the term *ventral* to denote a portion of the lateral nucleus and identified the midline nuclei (*Kern der Mittellinie*) [6]. Following Nissl's cell labeling discovery, his mentor Bernhard von Gudden realized its potential application for connection tracing [7]. Performing selective ablations of the cortex, he was able to detect morphological changes, gliosis, and atrophy in distant brain regions. Expanding on this experimental approach, Constantin von Monakow delineated a first general topology of thalamocortical connections [8] (Fig. 1J and K). This work particularly helped disambiguate ‘essential’ thalamocortical projections that degenerated after lesioning of circumscribed cortical areas from ‘sustaining’ projections, which required lesioning of multiple areas before they showed detectable degeneration [9]. This distinction provided first evidence that some thalamocortical projections may be distributed more diffusely to the neocortex than others.

The introduction of ‘Marchi's method’ in 1885 provided further insights into thalamic connectivity and their association with other subcortical structures [[10], [11], [12]]. This technique allowed anterograde staining of myelinated fiber bundles during post-lesion degeneration and was able to resolve thalamic termination sites of the main subcortical inputs [13]. The application of Marchi's method revealed a general *Bauplan* of the subcortical afferent system revealing consistent terminations of deep brain nuclei within thalamic territories without direct projections to the cerebral cortex. Furthermore, tracing of thalamic afferents allowed the distinction of individual tracts, including the spinothalamic and trigeminothalamic afferents, the auditory pathway, optic tract, cerebellothalamic tract (CTT), as well as the ansa peduncularis, and their associations with the broader functional networks [13]. These studies provided conclusive evidence, solidifying the role of the thalamus at the interface of sensory and motor integration.

Functionally, motor-related areas were viewed as relay stations until the 1980s. They were believed to transmit input from cerebellum and pallidum to cortex, with activity being exclusively controlled by inhibitory influence from the thalamic reticular nucleus and feedback from primary and supplementary motor areas. However, subsequent research in carnivores and non-human primates established a much more intricate circuitry, identifying a multitude of inhibitory interneurons that modulate the afferent inputs via dendro-dendritic and axonal synapses on thalamocortical projection neurons, known as glomeruli. The thalamocortical projections are in part reciprocated by strongly developed cortico-thalamic projections. Hence, the flow of information to the cortex is not passively relayed but, through the intermediary of interneurons, subjected to complex modulatory influences [[17], [18], [19], [20]].

**Fig. 2:**
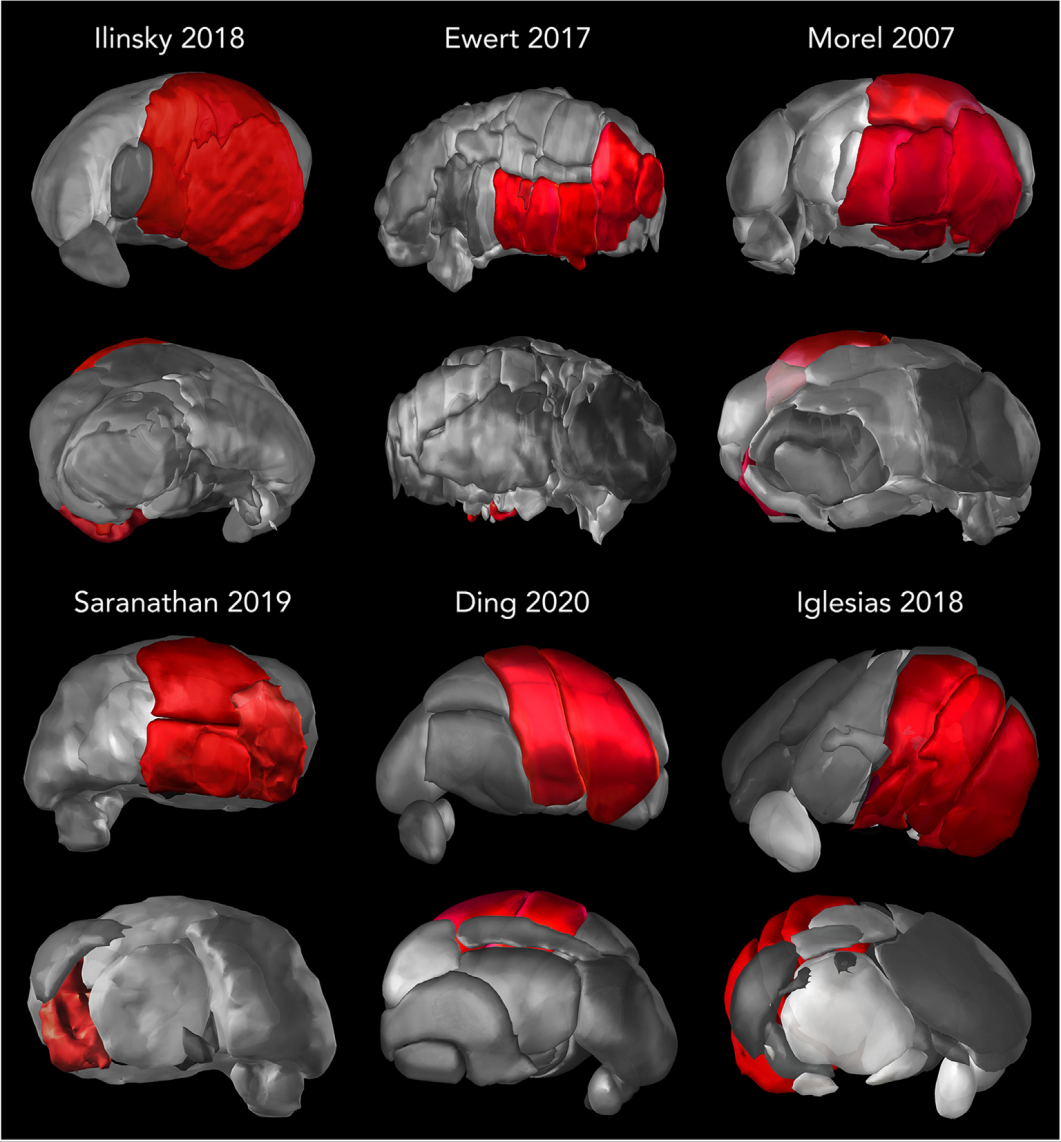
**Heterogeneity in the delineation of the motor thalamus across atlases.** The figure illustrates different parcellation schemes of the thalamus (in gray) and the motor thalamic nuclei (red), as defined by different anatomical atlases. The variability can in part be attributed to the diverse methods used to identify the boundaries of the thalamic nuclei and the translation of findings from non-human primates to human anatomy, in part by different levels of granularity in their segregations. The reconstruction by Ewert uses the Hassler nomenclature and features the motor thalamus as assigned by Hassler [31] capturing only the ventral portion of the thalamus. Notably, while the term ‘motor thalamus' typically refers to the territory occupied by cerebellar, pallidal, and nigral. Contemporary pathway tracing studies reveal that both pallidal and cerebellar projection zones span up to the dorsal edge of the thalamus, encompassing Hassler's dorsal nuclei. Historically, the emphasis on the ventral part is due lack of experimental data at the time and its significance as a neurosurgical intervention target. Atlases adopted from Ilinsky et al. 2018 [32], Ewert et al. 2017 [33] which is based on histological data from Chakravarty et al. 2006 [34], Morel 2007 [35], Saranathan et al. 2019 [36] (based on the Morel atlas), Ding et al. 2020 [37] and Iglesias et al. 2018 [38] (based on the Morel atlas) as available in Lead-DBS.

Driven by ever improving staining and labeling methods, it soon became evident that thalamic nuclei featured variable circuitry, hinting at different information processing mechanisms. As studies were conducted across different institutions in Europe and North America, this led to the proliferation of inconsistent delineations and terminologies [21] (Fig. 2). In humans, neuropathological studies and stereotactic atlases devised to guide thalamotomies particularly influenced the delineation and nomenclature of thalamic nuclei [1,[22], [23], [24]]. As a result, two large families of nomenclature are recognized today: 1) the ‘Anglo-American’ nomenclature which was originally proposed by Walker [25] based on his studies of cortex-thalamus connections in chimpanzees, adopted and expanded to the rhesus monkey by Olszewski [26], and revised and translated to humans by Hirai and Jones [2,27], and 2) the nomenclature offered by Hassler, which was adopted in the Schaltenbrand stereotactic atlas [1]. Hassler's work was fundamentally influenced by the myeloarchitectonic studies of the human thalamus as determined by Cécile and Oskar Vogt. Based on their original description, Hassler sought to improve and systematize the terminology dividing the thalamus based on its cortical and subcortical projection zones, dividing the nuclear lateral mass into up to 27 subdivisions and relating it to a stereotactic coordinate system [6].

### The controversy over the terminology of the human motor thalamus

The Hassler nomenclature of the motor thalamus is widely used in the neurosurgical community today, especially through the close connection with the Schaltenbrand Atlas; however, the terminology is not without criticism and has been called into question by several groups. Notably, Jones disputed Hassler's description of the ventro-oralis posterior (V.o.p) nucleus declaring that it “has no standing as an independent nucleus” [2]. He argued that Hassler made this distinction assuming differential afferent input into the ventro-oral nuclei of the thalamus. Specifically, Hassler thought that V.o.p received cerebellar projections, whereas the ventro-oralis anterior (V.o.a) nucleus received pallidal afferents [28]. However, according to Jones, both nuclei receive pallidal input rendering a distinction of the nuclei, at least from a functional perspective, obsolete. As a consequence, Hirai and Jones reconciled some of the thalamic subdivisions, merging, among other nuclei, V.o.a and V.o.p into a single nucleus, emphasizing the importance of functional connectivity over thalamic cytoarchitecture [27,29].

**Fig. 3:**
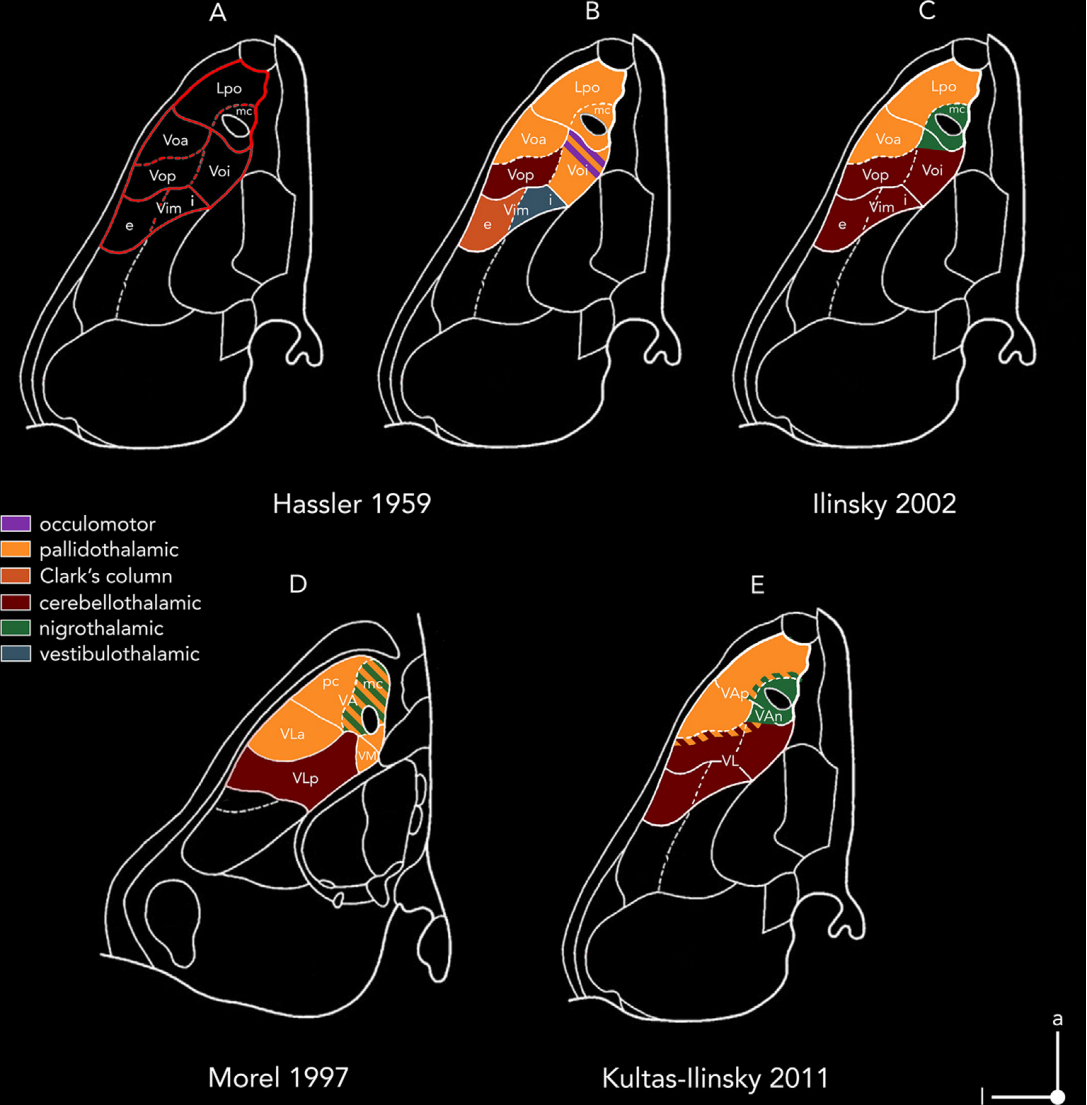
**Evolution of the understanding of subcortical afferent termination zones in the motor thalamus.** The figure features the chronological progression in understanding the topography of termination zones within the motor thalamus, focusing on the cytoarchitectonic confines outlined by Hassler **(A-C, E)** and Morel **(D)**. Outlines are shown in the axial plane 2 ​mm above the intercommissural line. Colors code for afferent termination zones of cerebellothalamic (dark red), pallidothalamic (orange), and nigrothalamic (green) projections as identified and revised by different groups. Hassler's description of thalamic termination zones additionally incorporates vestibulothalamic afferents (blue) projecting to Vim.i, ascending pathways for muscle spindle excitations originating in Clark's column (dark orange) that reach Vim.e, and oculomotor afferents projecting from the interstitial nucleus of Cajal to Voi **(A,B)** Nomenclature and zones as originally described by Hassler [16]. **I** Afferent termination zones extrapolated from tracing data in monkeys by Ilinsky et al. [39]. **(D)** Outlines from Morel's atlas, nomenclature introduced by Walker, which was revised and translated to humans by Hirai and Jones and adopted by Morel et al [25]. **I** Revised nomenclature based on immunocytochemical staining specific for afferent zones in human thalamus [32,40]. Adopted from Schaltenbrand and Wahren [1] (A-C,E) and Morel et al [35]. (D).

Percheron et al. extended this concept proposing an entirely new subdivision of the motor thalamus in accordance with the termination zones of globus pallidus, deep cerebellar nuclei, and medial/trigeminal lemnisci [30]. However, not all neuroanatomists agreed with this revised parcellation scheme. Recognizing the limitations of the Hassler nomenclature, Ilinsky and Kultas-Ilinsky proposed a reassignment of nuclei described by Hassler according to termination zones instead of redefining their borders (Fig. 3) [39].

According to them, the motor thalamus should be segregated into a pallidal zone including the latero-polar nucleus (L.po) and V.o.a (corresponding to the ventral anterior thalamic nucleus (VA)), and a cerebellar zone including V.o.p, V.i.m, and the posterior part of the ventro-oral nucleus, inner part (V.o.i), as well as Hassler's dorsal subdivisions [39] (corresponding to VL; Fig. 3C). Nigro-thalamic projections, which were largely neglected by Hassler were assigned to the magnocellular portion of the latero-polar nucleus (L.po.mc) and the anterior aspect of V.o.i [39]. Based on the availability of accurate immunocytochemical markers able to differentiate between the termination zones of the three subcortical afferents to the motor thalamus, this parcellation was further revised and amended in humans. This revised parcellation indicated that V.o.a predominantly received pallidal input, while V.i.m and V.o.p were primarily targeted by cerebellar input (for details refer to Table 2 and Fig. 3E) [40]. This work lends support to Hassler's original observations, partially reaffirming the distinctions he initially proposed.

In their revised parcellation scheme Ilinsky et al. followed the ‘Anglo-American’ naming convention introduced by Walker, labeling the entire basal ganglia projection zone as VA (Fig. 3E). Within this zone, they distinguished two subzones: VAp, which receives input from pallidum, and VAn, which receives nigral afferents [40]. The significance of nigral afferents to the primate thalamus was established by Ilinsky et al. [41], building upon their earlier description of nigral afferents in the cat thalamus [42]. This connection was largely ignored by Hassler and was absent from the nomenclature described by Hirai and Jones. The label VL (ventral lateral nucleus) was reserved for the cerebellar projection zone, which was further subdivided into ventral (VLv) and dorsal (VLd) territories based on the immunocytochemical marker SMI31 [32]. Importantly, the specific features and information processing within the three afferent zones (cerebellothalamic, pallidothalamic, and nigrothalamic) are reflected in their cytoarchitecture [[43], [44], [45], [46]]. This notion is in alignment with recent work by Mai et al. who called Hassler's extensive partitioning approach of the motor thalamus into question [21]. Their revised nomenclature assigned nigral territories to the medial ventral anterior nucleus (VAM), pallidal zones to the lateral ventral anterior nucleus (VAL), and cerebellar afferents to VL, analogous to the parcellation scheme used by Kultas-Ilinsky et al [40]. (Fig. 3E).

To comprehensively describe the thalamus and its constituent nuclei, two key components have emerged from historical research: 1) cytoarchitecture, which reveals the cellular organization and structure of the thalamus, and 2) connectivity, which assigns functional roles to the thalamic nuclei. To harmonize these aspects, Morel and colleagues translated the concepts of Hirai and Jones into a thalamic atlas based on human histological sections [35]. By applying a multi-architectonic parcellation scheme, combining Nissl, myelin, and immunohistochemical stains, these authors revised the boundaries of the thalamic nuclei. It is important to note, however, that while these stains helped disambiguate intralaminar and midline nuclei, they did not contribute much to the delineation of motor thalamic nuclei. Consequently, the latter are adaptations of the labeling used by Hirai and Jones. Nevertheless, the nomenclature introduced by Morel et al. was adopted by the International Federation of Associations of Anatomists (IFAA) and constitutes an international standard in the Terminologia Neuroanatomica today [47] (Fig. 3D, Table 1).

**Table 1:** Terminology of major nuclei and subdivisions of the human motor thalamus according to different authors and groups.

	Hassler 1959, 1977	Feremutsch & Simma 1971	Hopf 1971	Van Buren & Borke 1972	Hirai & Jones 1989; Jones, 1990, 1998	Percheron 2004	Morel 2007	Ding et al., 2016	TNA (2017)	Mai, Majtanik, 2018	Kultas-Ilinsky 2011, Ilinsky et al., 2018
**Motor Thalamic Nuclei**	L.po (mc), D.o.i in part	Rt.po, VA	L.po, L.po.mc, L.am, part of D (Dorsalkerne)	L.po, [D.a, V.o.anterior part]	VAmc (part of CeM), parts of VA	Vamc perifascicularis (=VOM = Voi)	Vamc	Vamc	Vamc	VAM, Vamc	VAn
	V.o.i, D.o.i in part		V.o.i.		VA, VLp anterior medial part	VA	V Apc ventral anterior parvocellular ncl.	Vapc	Vap		VL
	L.po.b (V.o.a)	VOM?	L.po.b	Fa	VMp		VM			VAMb	
	L.po.l, D.o.e	DA, VA	La.po	L.po, D.o, V.o.e	Part of VA, Vla, part of VLp	VO	VA, VLp			VAL=Vapr	VAp
	V.o.a.	VOA	V.o.a				Vla (Vap), VM, VA	VLR	Vla		
	V.o.p	DA	V.o	V.o.i		VLM/VlmM	VLpd			VLA	VL
	V.o.m	VOM	V.o.m		VM	VOM	VM	VM, VMb	VM, VMb, VMp	VALb	
	D.o.i	DA, DP	Part of D (Dorsalkerne)	D.o.	Part of VA and Vla	VA	VLp, part of VLpl		VLp	VAL-VLA-transition	
	D.im	DP	D.im		VLp, dorsal part	VIMps ​= ​Dips ​= ​VLps		VLC		VLP	
	V.o.i	VA, DA		V.o.i	VLp, antermedial part	VA					
	V.im.e	V.im, part of VOP	V.im.e	V.im.e	VLP, ventral part	VimL/VLL	Vla, VLpl			VLp?	
	V.im.i	VOM, part of VOP	V.im.i	V.im.i		VImM/VLM	VLpd, VLpv				

Abbreviations: CeM, Central medial nucleus; DA, ncl dorsalis anterior; D, nucleus dorsalis; D.im, Ncl. dorso-intermedius; Dips, Ncl. ventralis lateralis, (Situs postremus); D.o.e, Nucleus dorsooralis externus; D.o.i, Nucleus dorsooralis internus; DP, Ncl. dorsalis posterior; Fa, Nucleus fasciculosus; L.po, Nucleus lateropolaris thalami; L.po.b, Ncl. lateropolaris basalis; L.po.l, Ncl. lateropolaris lateralis; Rt.po, Nucleus reticularis Polaris; VA, Ventral anterior nucleus; VAL, lateral ventroanterior nucleus; VAMb, medial ventroanterior nucleus (basal part); Vamc, ventral anterior nucleus (magnocellular division); VAn, Ventral Anterior Nucleus, nigral part; VAp, Ventral Anterior Nucleus, pallidal part; Vapc, Ventral anterior nucleus (parvocellular division); V.im.e, Ventral intermediate nucleus, pars externa; V.im.i, Ventral intermediate nucleus of the thalamus, pars interna; VimL, Ncl. ventralis intermedius (pars lateralis); VImM, Ncl. ventralis intermedius (pars medialis); VIMps, Ncl. ventralis intermedius (Situs postremus); VL, Ventral lateral nucleus; VLA, Ventral lateral anterior nucleus; VLC, ventral lateral nucleus (caudal division); VLL, Ncl. ventralis lateralis (pars lateralis); VLM, Ventral lateral medial nucleus; VLp, Ventral lateral posterior nucleus; VLpd, Ventral lateral posterior nucleus (dorsal division); VLpl, Ventral lateral posterior nucleus (lateral division); VLps, Ncl. ventralis lateralis, (Situs postremus); VLR, Ventral lateral nucleus (rostral division); VM, Ventral medial nucleus; VMp, ventromedial posterior ncl.; V.o, Nucleus ventrooralis; V.o.a, Nucleus ventrooralis anterior; V.o.i, Nucleus ventrooralis internus; V.o.m, Nucleus ventrooralis medialis; V.o.p, Nucleus ventrooralis posterior; VO, Ncl. ventro-oralis; VOA, Ncl. ventralis oralis (pars anterior); VOM, Ncl. ventralis oralis (pars medialis). Adopted from Mai et al [21].

**Table 2:** Comparison of classifications of motor thalamic nuclei and their afferent zones in humans and rhesus monkey.

Afferent input	Rhesus monkey	Human		
	Ilinsky and Kultas-Ilinsky 1987, Ilinsky et al. 2002	Hirai and Jones 1989; Jones 2007	Kultas-Ilinsky 2011; Ilinsky et al. 2018	Hassler 1959
Nigrothalamic (SNr)	Vamc	Vamc	VA(n)	L.po.mc, parts of L.po and Doi
Pallidothalamic (al, fl)	Vapc, Vadc	VA, Vla, VM	VA(p)	V.o.a, marginally V.o.p, parts of L.po, D.o.i, V.o.i, Z.o, and D.o.e
Cerebellothalamic (CTT)	VL	VLp	VL (VLd ​+ ​VLv^a^)	V.im, V.o.p, and parts of V.o.i, V.o.m, D.im, D.o.i, Z.im, Z.o, and Z.c

Classifications were derived from the distribution patterns of subcortical afferents and cytoarchitecture as identified in the respective studies.

a VLv includes Hassler's V.im, V.o.p and aprts of V.o.i. Abbreviations: al, ansa lenticularis; DCN, deep cerebellar nuclei; D.im, dorsal intermediate nucleus; D.o.e, dorso-oral nucleus, external part; D.o.i, dorso-oral nucleus, internal part; fl, fasciculus lenticularis; L.po, lateropolar nucleus; L.po.mc, lateropolar nucleus, magnocellular part; SNr, substantia nigra, pars reticulata; VA, ventral anterior nucleus; VA(n), ventral anterior nucleus, nigral afferent zone; VA(p), ventral anterior nucleus, pallidal afferent zone; Vadc, nucleus ventralis anterior, pars densicellularis; Vamc, nucleus ventralis anterior, pars magnocellularis; Vapc, nucleus ventralis anterior, pars parvocellularis; V.im, ventral intermediate nucleus; VL, ventral lateral nucleus; Vla, ventral lateral anterior nucleus; VLd, ventral lateral dorsal nucleus; VLp, ventral lateral posterior nucleus; VLv, ventral lateral ventral nucleus; VM, ventromedial nucleus; V.o.a, ventro-oral nucleus, anterior part; V.o.i, ventro-oral nucleus, inner part; V.o.m, ventral-oral nucleus, medial part; V.o.p, ventro-oral nucleus, posterior part; Z.c, zentrolateral nucleus, caudal part; Z.im, zentrolateral intermediate nucleus; Z.o, zentrolateral nucleus. Adopted from Ilinsky et al [40].

**Table 3:** Overview of hotspots and targets derived from ET studies previously published in the literature.

Reference	Study Type	No. of patients	Follow-up period [Mo]	Coordinates (x/y/z) [mm]	Space	Outcome measure	Improvement [%]
Papavassilliou et al. [110]	Retrospective	37	26	−14.5/−17.7/−2.8	MNI	FTM (limited)	53.0
Hamel et al. [111]	Retrospective	10	12	−12.7/-7.0/-1.5	AC-PC	FTM (total)	70.1
Herzog et al. [112]	Prospective	10	18.4	−13.0/-5.5/0.0	AC-PC	Kinematic analysis	64.2
Blomstedt et al. [113]	Retrospective	21	12	−11.6/-6.3/-2.3	AC-PC	ETRS (total)	60.0
Barbe et al. [114]	Retrospective	21	3	−11.3/-7.2/-1.4	AC-PC	Kinematic analysis	65.0
Sandvik et al. [115]	Retrospective	17	66	−13.0/-1.8/4.1	AC-PC	ETRS (total)	48.4
Sandvik et al. [115]	Retrospective	19	12	−12.1/-5.5/-1.2	AC-PC	ETRS (total)	58.2
Fytagoridis et al. [116]	Prospective	50	12	−11.9/-6.2/-2.0	AC-PC	ETRS (total)	59.5
Cury et al. [117]	Retrospective	38	12	−14.7/7.1/1.8	AC-PC	FTM (total)	66.0
Fiechter et al. [118]	Retrospective	12	5–22	−14.3/-5.0/0.9	AC-PC	FTM (total)	54.0
Barbe et al. [119]	Prospective	13	12	−11.0/-5.7/-1.9	AC-PC	FTM (total)	64.0
Nowacki et al. [120]	Retrospective	21	12	−10.6/-5.2/-3.2	AC-PC	FTM (total)	55.0
Nowacki et al. [121]	Retrospective	15	12	−12.8/-3.6/0.0	AC-PC	Bain and Findley Score	63.0
Philipson et al. [122]	Retrospective	26	12	−12.0/-7.5/-4.0	AC-PC	ETRS (total)	63.0
Tsuboi et al. [123]	Retrospective	97	12	−14.3/-4.3/-2.1	AC-PC	FTM (total)	53.2
Elias et al. [124]	Retrospective	39	16.8	Probabilistic map	MNI	FTM (total)	42.8
Tsuboi et al. [125]	Retrospective	20	6.6	−15.0/−17.0/1.0	MNI	FTM (total)	38.7
Middlebrooks et al. [126]	Retrospective	84	6.8	−15.5/−15.5/0.5	MNI	FTM (total)	54.6
Nowacki et al. 2022	Retrospective	119	12	Probabilistic map	MNI	FTM (total)	64
Neudorfer et al. 2022	Retrospective	65	12	Probabilistic map	MNI	FTM (total)	65.1

Literature derived AC-PC coordinates were converted to MNI space as previously described [127] and are featured in Fig. 7 in relationship to motor thalamus and posterior subthalamic area. ETRS essential tremor rating scale; FTM, Fahn-Tolosa-Marin tremor rating scale; MNI, Montreal Neurological Institute.

### The motor thalamus in the context of movement disorders

The accurate description and delineation of the motor thalamus is crucially important in the context of stereotactic surgery where nuclear boundaries may guide surgical procedures. Based on theoretical grounds, Hassler himself first described the motor thalamus as a target in Parkinson's Disease (PD) [48]. He proposed that the optimal target for tremor control was not Vim, but Vop, which comprised his presumed cerebellar afferents. Remarkably, satisfactory tremor suppression could be achieved during ablation of the nucleus, even when Vim was spared as evidenced by postmortem examination [49]. Surprisingly, while lesions restricted to Vim also showed some effectiveness in treating tremors, the impact was less pronounced. This finding aligns with Hasslers understanding of Vim's role as a sensory relay nucleus, processing proprioceptive input from Ia muscle spindles (Vim.e) and vestibular information (Vim.i) (Fig. 3B) [1], but contradicts contemporary targeting approaches for essential tremor that specifically target Vim for best tremor control. To understand this discrepancy, it is crucial to consider potential limitations that Hassler faced when identifying thalamic subnuclei on autopsy material. It is also essential to acknowledge potential disruptions of fibers of passage, specifically the CTT, which were likely implicated in lesions targeting the base of the motor thalamus [50]. Hassler fully acknowledged the latter and this notion even formed the theoretical foundation for the subsequent adoption of ‘subthalamotomies’ by the German stereotactic school of Freiburg. These ‘subthalamotomies’ were often combined with small lesions in Voa and Vop to enhance treatment outcomes [51]. To avoid confusion with the present context we must emphasize that these ‘subthalamotomies’ did not target the subthalamic nucleus but were placed in the zona incerta and posterior subthalamic area (PSA) below the thalamus, sparing the subthalamic nucleus out of fear of inducing hemiballism [52]. Recent approaches for lesioning the subthalamic nucleus with focused ultrasound surgery, described as ‘subthalamotomies’ are hence not comparable and introduce a potentially misleading nomenclature in regard to historical context [53].

**Fig. 4:**
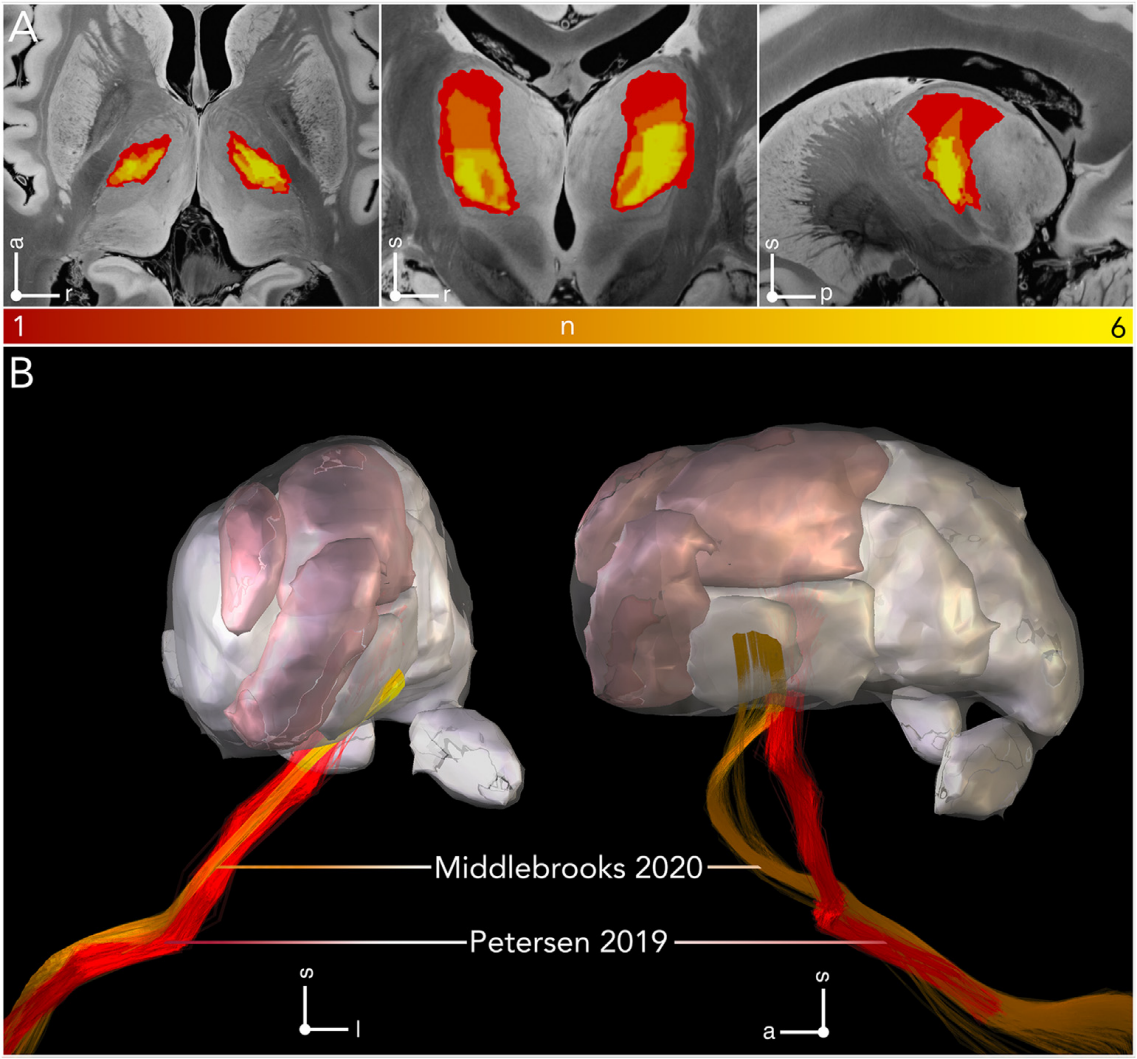
**Variability in the delineation of V.im/VL and the cerebellothalamic afferent system. (A)** N-map of Vim/VL parcellations, derived from the atlases in Fig. 2 is shown in axial, coronal, and sagittal planes, superimposed on a 100 ​μm resolution, 7T brain scan in MNI 152 space. The maps were generated by summing the binary atlas parcellations in a voxel-wise manner. **(B)** Passage of the cerebellothalamic tract as derived from different anatomical atlases. Tracts are featured exhibit significantly greater variability in their passage in the sagittal plane (right), as compared to the coronal plane (left) at the subthalamic level. Note that tracts converge onto V.im and are constrained with respect to their termination zones (refer also to Table 2). Adopted from Petersen et al. 2019 [64] and Middlebrooks et al. 2020 [65].

**Fig. 5:**
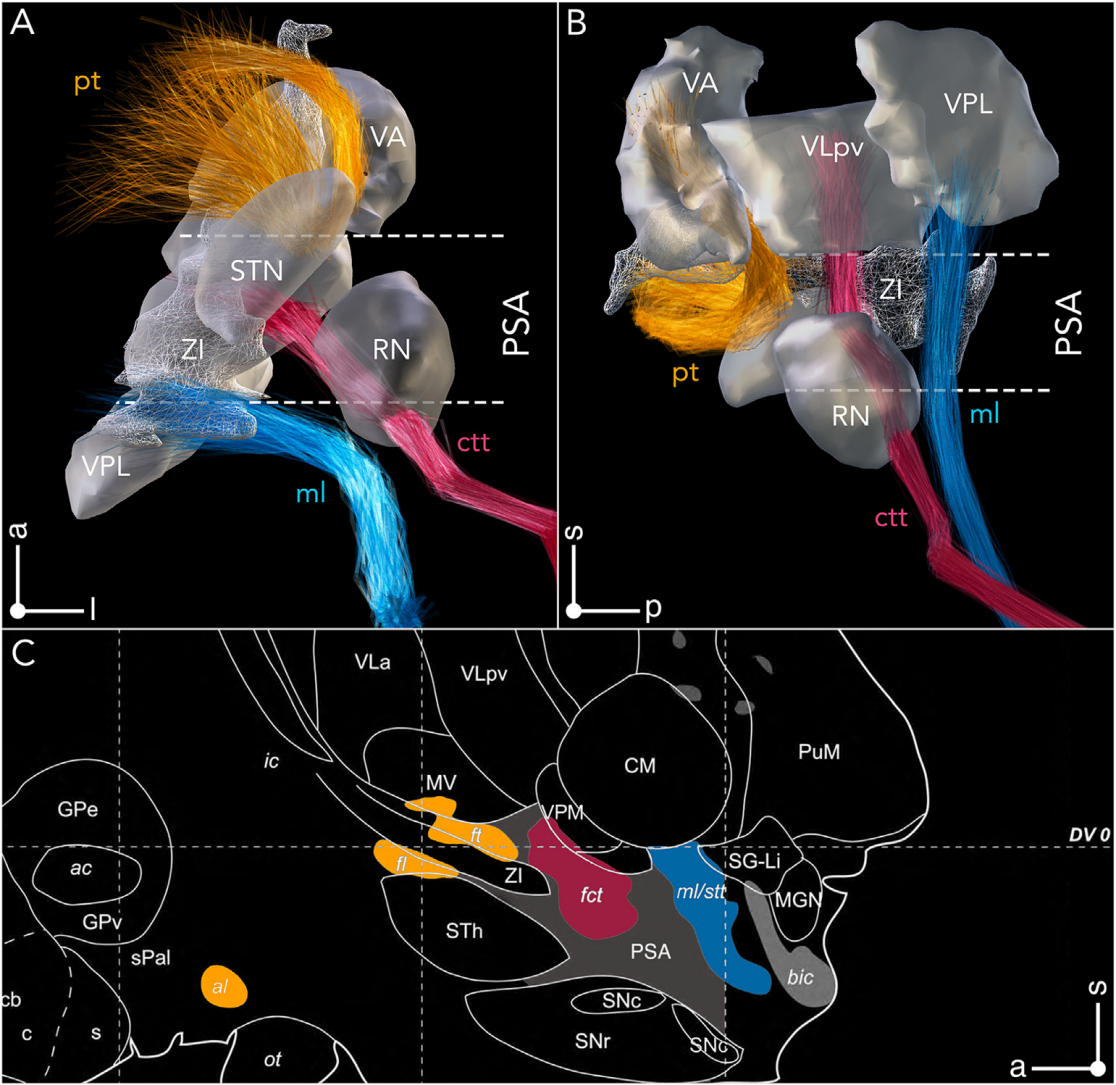
**Anatomical description of the posterior subthalamic area (PSA). (A)** Inferior (from below), **(B)** medial, and **(C)** sagittal views of PSA and its anatomical confines. Pallidothalamic fibers constrain the PSA anteriorly and traverse the internal capsule through ansa lenticularis (al, orange) and fasciculus lenticularis (fl, orange) forming the fasciculus thalamicus (ft, orange) medial to the STN, which ascends into the anterior nuclei of the motor thalamus. PSA adjoins the posterior border of STN. The caudal border of PSA is formed by the medial lemniscus (ml, blue) and spinothalamic tract (stt, blue), which ascend into the sensory thalamic nuclei. The main structures comprised within the PSA are zona incerta (ZI), prelemniscal radiation, which consists of fibers ascending from the mesencephalic reticular formation (not shown), as well as the cerebellothalamic tract (fct). Abbreviations: bic brachium of the inferior colliculus; CM centromedian thalamic nucleus; fct fasciculus cerebellothalamicus; fl fasciculus lenticularis; ft fasciculus thalamicus; ic internal capsule; MGN medial geniculate nucleus; ml medial lemniscus; PuM, medial pulvinar; PSA posterior subthalamic area; pt, pallidothalamic tract; SG suprageniculate nucleus; SNc substantia nigra, pars compacta; SNr substantia nigra, pars reticulata; STN nucleus subthalamicus; stt spinothalamic tract; VLa ventral lateral anterior thalamic nucleus; VLpv ventral lateral posterior nucleus, ventral portion; VM ventral medial nucleus; VPM ventral posteromedial nucleus. (C) adapted from Morel et al [35].

A main conclusion from Hassler's postmortem studies was that i) neuromodulation of Vim proper is not a prerequisite for improvement of tremor symptoms, ii) stereotactic interventions in nuclei outside of Vim may prove equally beneficial, and iii) involvement of CTT is crucial for optimal outcome. Indeed, although the pathophysiology of tremor generation has not been fully established to date, major importance has been attributed to modulating the cerebellothalamic system in the context of parkinsonian [[54], [55], [56]] and non-parkinsonian tremor [[57], [58], [59], [60]]. While the passage of CTT through the PSA is well established today [61], the tract also comprises extensive interdigitations with adjacent afferent territories (Fig. 4, Fig. 5). Specifically, CTT passes through somatosensory and intralaminar nuclei in the posterior thalamic region while intersecting with pallidothalamic afferents anteriorly, reaching the motor nuclei via the external medullary lamina [62,63]. Notably, while cerebellar and pallidal territories feature extensive interdigitations, the two systems remain separate at the neuronal level, with no convergence onto individual neurons having been reported from neuronal tracing studies.

### Relevant afferents to the motor thalamus and their pathways through the subthalamic area

The extensive coverage of the PSA by CTT may explain the efficacy of historical lesions across various regions of the motor thalamus, especially when targeting the ventral borders of thalamic nuclei. This interdigitation, however, also means that the exclusive modulation of a single afferent zone during surgical intervention is effectively not possible. Indeed, several white matter pathways intersect with cerebellar afferent territories, including – most prominently – pallidothalamic and nigrothalamic pathways, as well as fibers ascending from the mesencephalic reticular formation and pedunculopontine nucleus (Schaltenbrand and Wahren consolidated all these tracts as the prelemniscal radiation (Raprl) [66]). Furthermore, the zona incerta (ZI) is closely associated with these territories and optimal tremor outcome has been associated with stimulation of this structure [67].

### The electrophysiologically defined Vim

In addition to the anatomical definition of the motor thalamus, electrophysiological recordings have been instrumental in identifying the optimal tremor target. The research group led by the electrophysiologist Denise Albe-Fessard and the neurosurgeon Gerard Guiot in Suresnes, a suburb of Paris, provided the first evidence for the discrimination of thalamic nuclei by means of microelectrode recordings [68]. Their innovative implantation and recording methods facilitated the distinction of electrical activity patterns in the pulvinar, sensory, and motor thalamic nuclei. This allowed them to map the topographical arrangement of the sensory thalamus and to differentiate distinct neural populations based on both spontaneous activity and evoked potentials [69].

**Fig. 6:**
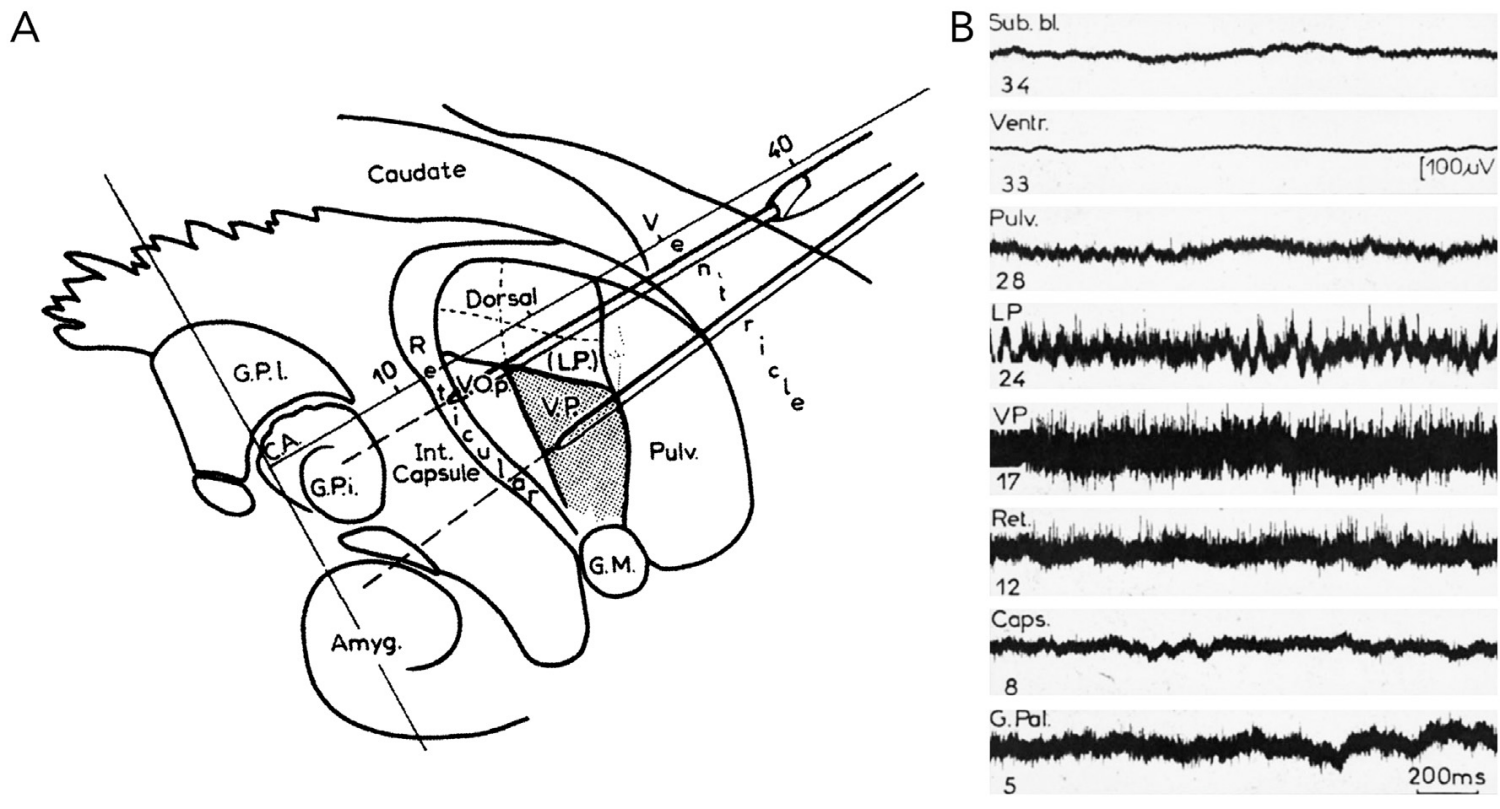
**The ‘electrophysiologically defined V.im’ as pioneered by Albe-Fessard and Guiot. (A)** Sagittal section through the thalamus, showing two of the most commonly chosen trajectories for stereotactic lesioning by the group using a parietal approach. At the thalamic level, the trajectory typically traversed pulvinar (Pulv.), nucleus lateralis posterior (L.P.), the superior part of the nuvleus ventralis posterior (V.P.), and nucleus ventralis lateralis (defined as V.O.p by Albe-Fessard et al.). **(B)** Differentiation of thalamic nuclei based on oscillographic recordings. During thalamic exploration, spike activity intensifies in the nucleus lateralis posterior (LP) and is especially prominent in the nucleus ventralis posterior (VP). This activity diminishes in the nucleus reticularis and is significantly reduced in the internal capsule. Entering the globus pallidus (G.P.i.) leads to another spike activity surge. Adopted from Albe-Fessard et al [75].

One of the group's most significant contributions was the identification of tremor-synchronous bursting cells which led to the establishment of the “electrophysiologically defined Vim” (Fig. 6). Microelectrode recordings in patients suffering from tremor revealed a posterior to anterior gradient of functional organization featuring neurons responsive to light touch most posteriorly in the sensory thalamus (tactile Vc) [[70], [71], [72], [73]]. Anterior to this region was a territory responsive to proprioception, that then transitioned into an area associated with passive movement and activation of muscle spindles. The cells in this territory have been characterized as kinesthetic neurons and feature a rhythmic discharge pattern that is time-locked to a patient's tremor frequency. While this region encapsulates parts of Vim proper as defined cytoarchitectonically by Hassler, it is important to note that this area is not synonymous with Vim. Rather, the “electrophysiologically defined Vim” would map to a region comprising the anterior border of Vim and posterior border of Vop [63]. Intraoperative high-frequency stimulation of this region has been associated with optimal tremor suppression [74]. During the lesional era, low-frequency stimulation was frequently applied to confirm accurate electrode placement. It was reported that the effect of stimulation on thalamic neurons changed from posterior to anterior, where low-frequency stimulation posteriorly induced increases in spike amplitude concomitant to tremor activation, whereas anterior stimulation reduced or eliminated tremor [76,77]. Cells anterior to the “electrophysiologically defined Vim” include neurons preferentially responding to active rather than passive movements [78]. Electrolytic lesions in the posterior region were ultimately chosen as the target since they resulted in long-lasting positive clinical effects on tremor.

### The demise of PSA during the advent of the modern era of DBS

The electrophysiological characterization of Vim and its role in tremor suppression laid the foundation for the modern era of thalamic targeting in movement disorders. This period was characterized by a pronounced emphasis on Vim proper as the optimal substrate for tremor control and sidelined neurosurgical intervention in the posterior subthalamic area (PSA) as promoted by Hassler and the German stereotactic school of Freiburg. This preference can be attributed to the relative ease by which Vim could be delineated using microelectrode recordings [67]. It has also been suggested that the PSA was abandoned because of side effects and complications associated with procedures in this region [57,58]. However, this perspective is not universally supported by the existing literature [67]. Another contributing factor might be that many surgeons, influenced by the pioneering work of Benabid et al [79,80]. predominantly described Vim proper as their preferred target. Benabid's target selection also influenced the first multicenter, randomized-controlled trials which were instrumental in the approval of Vim-DBS as a therapy for ET and PD in Europe and Canada in 1993, followed by FDA approval in 1997 [81]. These trials evaluated the efficacy of unilateral Vim-DBS over periods of 12 and 34 months in cohorts of n ​= ​83 and n ​= ​19 patients, respectively, demonstrating a clear reduction in action (intention and postural) tremor scores in the DBS-ON vs DBS-OFF state and finally lead to FDA-approval of this therapy (Fig. 9). Of note, the overall number of side effects (temporary and permanent) reported for ET patients in these trials was 55 ​% as compared to 30 ​% in PD patients, with paresthesia and dysarthria being the most common [81]. Despite neglect of PSA in anatomical discussions during the early days of modern DBS, its close proximity to the Vim target and the tremor cells at the ventral thalamic border suggest that these procedures stimulated thalamic afferents, especially the CTT. Indeed, deeper electrode placements and stimulations at high intensities would have directly affected thalamic afferents in PSA. Early uncontrolled studies using the first generation Medtronic and Avery Laboratories stimulators even hinted at the potential superiority of PSA in tremor control [82,83] with uncontrolled evidence of long-term efficacy [84]. However, at the time, this research was not given the attention it may have deserved.

**Fig. 7:**
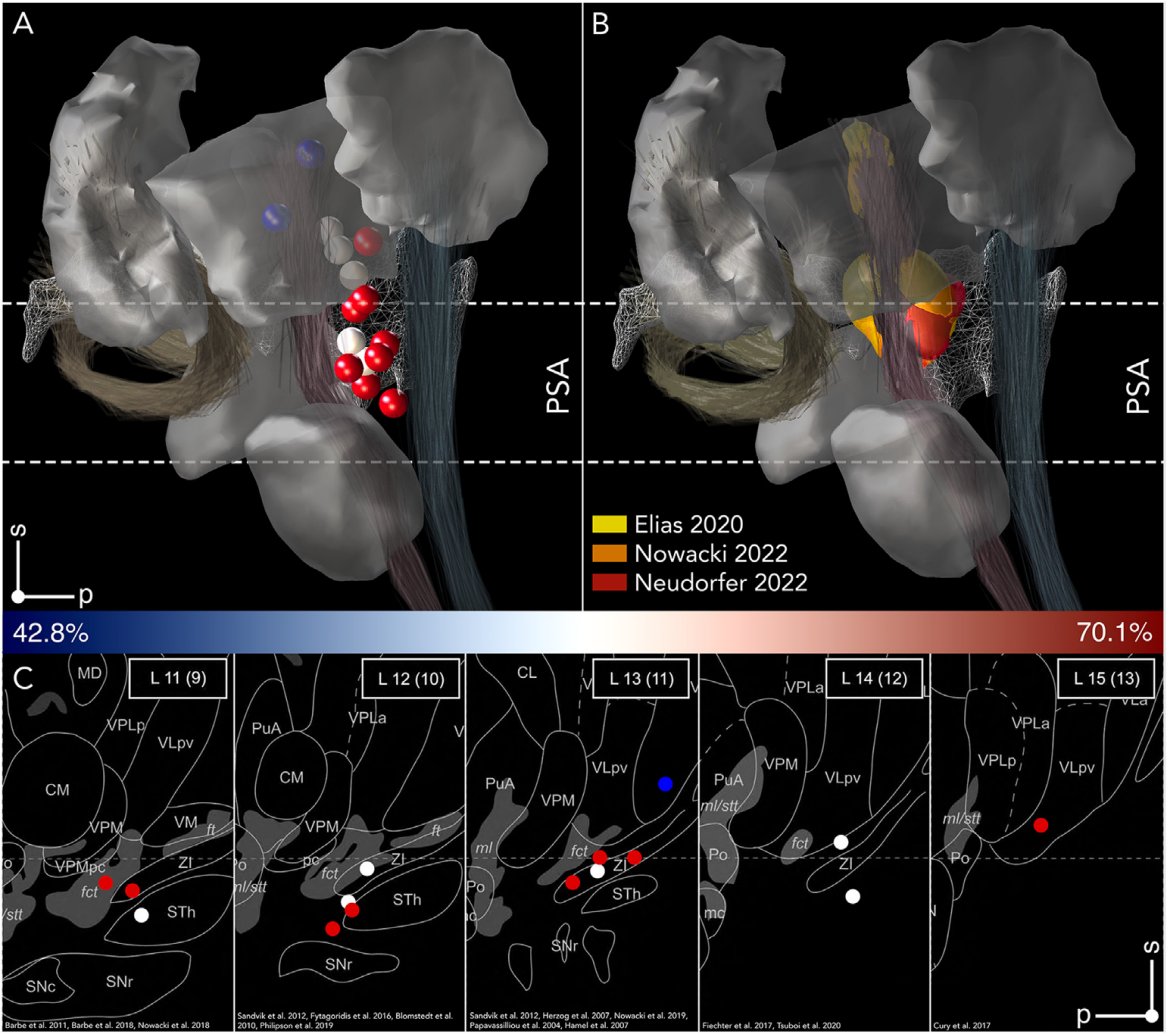
**Spatial relationship between anatomical structures and previously reported DBS targets in the literature. (A)** Medial view of the thalamus and posterior subthalamic area (PSA) features previously reported target coordinates associated with symptom improvements following DBS of the V.im and PSA. Targets associated with improved clinical outcome are closely related to the cerebellothalamic outflow tract (dark red) and the zona incerta (white mesh) at the level of the PSA. For a comprehensive overview of study characteristics, MNI coordinates, and clinical outcomes, see Table 3. **(B)** Relationship of publicly available DBS hotspots to anatomical substrates. Probabilistic maps were derived from large cohorts of ET-patients undergoing DBS and are featured in 3D. **(C)** Sagittal sections feature previously reported target coordinates within PSA and motor thalamus. Mediolateral planes, expressed in millimeters relative to the intercommissural line and estimated from the margin of the third ventricle (indicated in parentheses), are specified in the upper right corner of each section. Sections are aligned with the intercommissural line (horizontal dashed line).

**Fig. 8:**
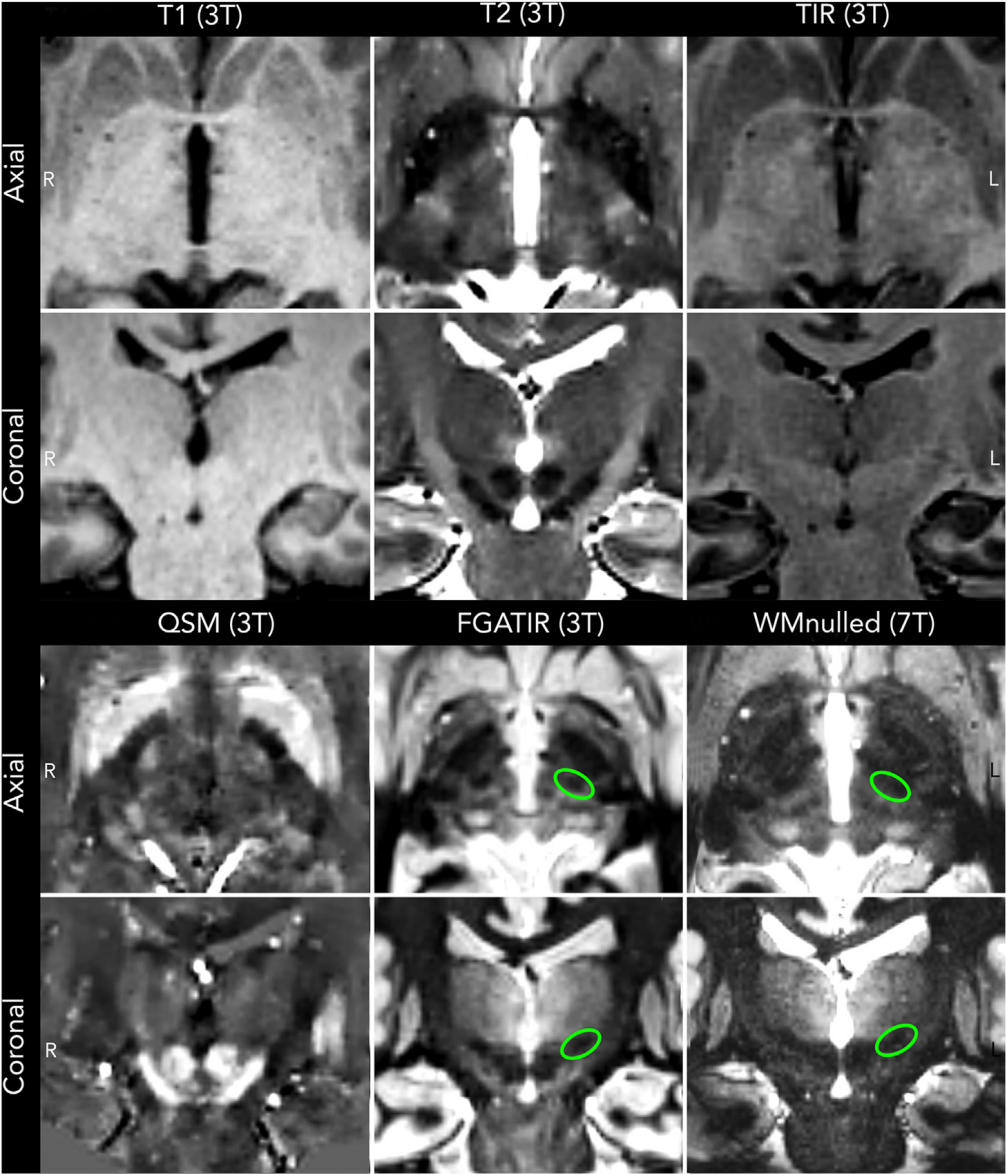
**Comparison of conventional and advanced imaging sequences used for surgical planning in V.im-DBS.** Top row: Conventional imaging sequences, including T1w-imaging, T2w-imaging, and inversion recovery sequences have been clinically effective, but lack of contrast at the thalamic level, which hinders the direct identification of the DBS target and surrounding anatomical substrates during surgical planning. Bottom row: Advanced imaging sequences such as quantitative susceptibility mapping (QSM) and white-matter nulled (WMnulled) sequences (e.g., fast gray-matter acquisition T1 inversion recovery (FGATIR) sequences), provide superior contrast at the thalamic level allowing the identification of individual thalamic nuclei and white matter tracts, which can be used to tailor the target towards the patient's anatomy. Increases in magnetic field strength enhance the signal-to-noise-ratio and yield improved resolutions, however, they also have distinct disadvantages including increased sensitivity to motion and susceptibility artifacts. Green circles denote a hypointensity at the level of the PSA as identifiable on FGATIR and WMnulled sequences, considered to be the imaging equivalent of the cerebellothalamic tract (CTT). Overlap of stimulation volumes with the hypointensity was shown to predict clinical outcome in patients undergoing DBS for ET.

**Fig. 9:**
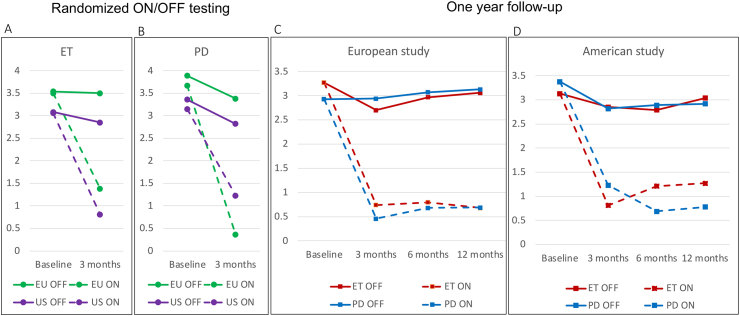
**Efficacy data available to the FDA for the approval of Vim-DBS of the first multicenter, uncontrolled trials for therapy for ET and PD in Europe and North America. (A, B)** Reduction of mean tremor scores (0–4) contralateral to the stimulated side in a randomized ON/OFF design as the main outcome parameter of the two studies at 3 months after implantation. This outcome was measured for 16 ​ET-patients and 13 PD-patients in the US-study and for 6 Patients with ET and 9 patients with PD in the EU study, respectively. **(C, D)** Open one-year follow-up of tremor improvement during open ON- and OFF-assessments for essential tremor (EU n ​= ​28 patients; USA n ​= ​45) and PD (EU: n ​= ​57; USA n ​= ​39). The graphs illustrate how the criteria for the approval of such therapies was handled at the end of the last century. Adopted from Food and Drug Administration (FDA) [81].

### From Vim to the Vim/PSA controversy in essential tremor DBS

Today, the ideal neuroanatomical target for tremor control remains a subject of ongoing debate, despite its extensive historical background and broad clinical application. This ambiguity arises from several factors: First, the nuclei of the motor thalamus cannot be clearly discerned on conventional imaging making it challenging to identify the gray/white matter boundary between thalamus and PSA. Second, the ultimate electrode location and the regions influenced during chronic DBS may extend beyond the confines of the electrophysiologically defined Vim during microelectrode recordings. Finally, targeting of PSA lacks standardization. Consequently, depending on the coordinates and trajectory employed, the final target may feature considerable variability across centers with at least four different regions currently under scrutiny (Fig. 7) [67]. This variability in an area larger than the STN complicates direct comparisons.

### Is the PSA a better target for tremor control with DBS?

Following the FDA's endorsement of Vim DBS, localization studies began to emerge seeking to pinpoint the most effective stimulation sites for tremor suppression. Based on earlier observations of lesional surgery [83,85,86] the Kiel group provided a systematic account of Vim vs. PSA stimulation, retrospectively analyzing DBS lead coordinates in a total of ten patients [87] followed by assessment of a larger group of patients confirming the superior effects of subthalamic versus Vim stimulation with different tremors [86]. They found that stimulation at ventral contacts generally outperformed dorsal contacts, with improvements exceeding 75 ​% typically occurring at or below the intercommissural line (ICL). The primary substrates associated with optimal outcome were Raprl and ZI. A subsequent kinematic analysis by the same group revealed that stimulation below the thalamic border, as identified using MER, resulted in a 99 ​% reduction of the accelerometry total power, in contrast to a 68 ​% reduction with ventral thalamic border stimulation and a mere 2.5 ​% reduction with stimulation within thalamus proper [86]. These findings were consistent during reach-to-grasp movements. Several small prospective studies corroborate these findings reporting tremor reductions of 60–81 ​% during PSA-DBS [54,58,88,89]. Similarly, a retrospective analysis by Blomstedt et al. on a larger cohort (n ​= ​68) indicated that 89 ​% of ET patients experienced improved hand function with PSA-DBS, compared to 70 ​% with Vim-DBS [90]. Improved tremor control has also been associated with PSA-DBS when using standardized and optimized stimulation protocols [87,91]. Retrospective evaluations have consistently shown that electrical stimulation inferior to the intercommissural line is more effective than superior to it, both in the short (up to two years) [92,93] and long term (four years or more) [94,95]. Eisinger et al., however, reported contradicting findings noting superior tremor suppression in Vim during long-term follow-up in 47 ​ET patients [93]. They also highlighted that Vim-DBS required lower stimulation intensities for adequate tremor control three years post-lead implantation (2.55 ​V vs. 3.17 ​V). Limitations of this study include its retrospective character and the predominance of unilateral implantations in the study. Thus, axial tremors were likely less well controlled which complicates generalizability of results. which complicates generalizability of results.

Meanwhile, two notable trials have compared the effects of Vim-DBS and PSA-DBS within individual subjects. A small study by Chang et al. explored the stimulation effects of Vim, PSA, and a combination of both in five ET patients, but found no significant differences in tremor improvement across these conditions [96]. Conversely, Barbe et al. conducted a randomized, double-blind, crossover trial in 13 ​ET patients [97]. Leads were strategically positioned to ensure individual contacts within Vim, ICL, and PSA. After an initial three-month stimulation at the ICL level, patients were randomized to stimulation either at Vim or PSA contacts. DBS was maintained for two months at these settings and then switched to the alternate contact for another two months. The study concluded with a seven-month period allowing for flexible adjustment of stimulation parameters based on clinical presentation. While no statistically significant differences in tremor control were found between the two targets, there was a trend towards better symptom suppression with PSA-DBS in the blinded period. Both groups had comparable side-effect profiles. In the open-label phase, 64 ​% of patients were stimulated in the PSA, 21 ​% in the Vim, and 7 ​% at the intercommissural level, with the remainder using complex stimulation paradigms. Interestingly, Vim-DBS required significantly higher stimulation intensities than PSA-DBS for optimal tremor control (5.88 ​mA vs 4.35 ​mA, p ​= ​0.0006). The relatively high stimulation amplitudes in both cohorts, however, suggest that the lead placements may have deviated from the typical Vim and PSA targets.

### Local mapping of DBS responses in essential tremor

The introduction of stimulation volume modeling and group-level analysis in the context of local- and network-mapping have greatly facilitated the identification of anatomical substrates associated with clinical improvement following DBS [98]. Dembek et al. performed the first association of clinical outcome with stimulation volume location in patients with ET [99]. Mapping 600 monopolar reviews in 16 patients the authors identified an optimal target for suppression in PSA that encroached on the inferior border of Vim. Subsequent probabilistic mapping studies provided further evidence that stimulation within PSA may be superior for control of tremor symptoms [[100], [101], [102], [103], [104], [105]]. Of these studies, Nowacki et al. conducted the largest retrospective analysis to date investigating changes in tremor control in a total of 119 patients. Their hotspot covered large parts of the motor thalamus as well as PSA, with peak intensities of optimal tremor control extending from posterior PSA to central Vim. Suboptimal outcomes mapped to the anteromedial aspect of PSA and Vop. A Few studies have reported contrary findings, identifying hotspots associated with optimal tremor improvement in the motor thalamus proper [[106], [107], [108]]. In their study of 33 ​ET patients, Elias et al. identified a hotspot predictive of outcome in the anterodorsal border of Vim, encroaching on Vop [107]. Interestingly, their coldspot (least correlated with tremor control) was located posteroventrally within ZI and ventral Vim. Of note average outcome in this cohort was 42.8 ​% as assessed using the Fahn-Tolosa-Marin Tremor Rating Scale (FTM), which is lower than typically reported in the ET-DBS literature [102]. Nordin et al. described a trend towards greater improvement within a hotspot covering the ventral borders of VLpv, VM, and VPM noting a trend towards symptom worsening in more distal contacts during monopolar review [106]. Stimulation within this region, however, was associated with a need to increase stimulation amplitudes for optimal symptom suppression. Hotspots generated using clinical settings revealed a ventral shift of peak improvement towards the ventral border of VLpv (Fig. 7, Table 3).

### Network mapping of DBS responses in essential tremor

The majority of probabilistic mapping studies support the notion that the PSA is the optimal target for optimal tremor control. This conclusion is supported by summarizing the main findings of the groups in Freiburg in the 60-ies, Montreal in the 70-ies and the later DBS-experience of the groups in Umea, Kiel, Cologne and the recent large multicenter-assessments. Recent additional evidence appeared in three studies published in the same issue of Annals of Neurology in 2022 [102,104,105], each of which applied modern neuroimaging techniques and unanimously favored the PSA, as summarized in an editorial [109]. Moreover, while peak stimulation locations associated with peak outcome feature notable variation across studies, most if not all of them appear to fall along CTT (Fig. 7). This observation aligns well with the notion that the target for tremor is connected to a pathological circuit relaying oscillatory activity between motor cortex, pontine nuclei, cerebellum, thalamus and back to motor cortex [[128], [129], [130], [131]]. Indeed, several connectivity studies highlight the CTT, whose involvement in tremor suppression constitutes the most accepted concept in connectomic DBS today. Using patient-specific DWI, Klein et al. demonstrated strong connectivity between motor thalamus and M1, demonstrating robust cerebellar projections through the superior cerebellar peduncle in patients undergoing Vim DBS [132]. In a randomized, crossover trial Dembek et al. were able to explain differences in tremor improvement based on the distance to CTT [133]. While this study relied on normative structural connectivity, subsequent work by the same group using probabilistic tractography in individual patients confirmed that effective outcomes in ET were located in close proximity to the CTT and that contacts in the PSA were overall closer to the tract compared to contacts within Vim proper [134]. Studies employing patient-specific [135], population- [103,108,136], and atlas-based [137] reconstructions of the CTT arrived at similar conclusions and served as the impetus for adopting these approaches in clinical practice [[138], [139], [140], [141]].

A few structural connectivity studies identified networks other than the cerebellothalamic system for optimal tremor control [[142], [143], [144]]. These studies identified a network connecting the thalamic ventral-oralis complex to premotor cortex (PMC) and supplementary motor area (SMA) to predict tremor improvement following DBS. It is important to note, however, that the study by Pouratian et al. harbored several limitations including small sample size (n ​= ​6), missing stimulation volume calculations, poor angular resolution, and variability in connectivity-based segmentations across patients [142]. While Middlebrooks et al. addressed many of these limitations, they arrived at the same conclusion [143]. Given that both studies employed comparable methodologies and connectivity measures, the potential for systematic bias exists. Finally, Nowacki and colleagues sought to identify the optimal tracking algorithm for reconstruction of the cerebellothalamic tract. Based on four different methodologies the authors noted significant anatomical variability. The Euclidean distance between active contact and the center of the tracked cerebellothalamic tract showed no significant correlation with clinical outcome (n ​= ​6) [144].

Functional connectivity is understudied in comparison to structural connectivity in ET DBS. Using a normative functional connectome to seed BOLD signal fluctuations from stimulation volumes in 36 ​ET patients, Al-Fatly et al. were able to estimate tremor improvements based on an identified network encompassing among others M1, sensory cortex, superior, and inferior cerebellar lobules [145]. Structural connectivity analysis was largely concordant with functional connectivity findings. Similar results were reported by Tsuboi et al. who investigated differential network profiles relevant for treating ET vs dystonic tremor [108]. Relying on patient-specific fMRI data acquired in ten patients during active Vim DBS, Gibson and colleagues reported activation of established nodes in the tremor circuit, namely sensorimotor cortex, thalamus, cerebellar cortex, and deep cerebellar nuclei [146]. Stimulation-induced activation of this network, as well as activation of SMA, brainstem, and inferior frontal gyrus correlated significantly with long-term improvement of tremor symptoms. Importantly, occurrence of stimulation-evoked side effects correlated with activation in pre-, post-, and subcentral regions.

The current literature underscores the involvement of broad cortico-basal ganglia and cortico-cerebellar networks in the pathophysiology and management of ET. This growing recognition of ET as a network disorder opens up the possibility for effective modulation of the tremor network at sites beyond Vim and PSA. Indeed, efficacious suppression of ET symptoms has been demonstrated through stimulation of the subthalamic nucleus [147] and deep cerebellar nuclei [148]. Despite these promising findings, however, the predominant choice for ET treatment remains Vim/PSA, based on extensive clinical experience and established efficacy. Surgical targeting strategies are well established, regions associated with optimal symptom control and side-effects have been thoroughly mapped, and effective stimulation paradigms are well recognized. While exploration of alternative, yet potentially equally effective targets has been limited to date, the field's now comprehensive understanding of Vim/PSA targeting may encourage the principled investigation of other approaches enabled by modern analytic techniques.

### The imaging-derived Vim

Magnetic resonance imaging (MRI) has evolved as the gold-standard for Vim targeting today [149]. However, the majority of DBS centers rely on conventional imaging sequences, such as T1w and T2w imaging for surgical planning, but lack of gray-white matter contrast at the thalamic level hinders the direct identification of thalamic targets and surrounding anatomical substrates during surgical planning [149]. Consequently, surgeons often rely on indirect targeting approaches for Vim. These approaches estimate the surgical target in relation to identifiable landmarks, such as the Anterior Commissure-Posterior Commissure (AC-PC) plane and the border of the third ventricle, but may not adequately consider inter-individual anatomical variability, necessitating the development of methods to target Vim more reliably and effectively.

Recognizing the role of CTT in tremor control and based on a growing appreciation for interindividual anatomical variability, concerted efforts have been directed towards the visualization of the cerebellothalamocortical network in recent years [150,151]. dMRI in particular has emerged as a pivotal tool for visualizing CTT, guiding patient-specific targeting, and assessing surgical outcomes [145,150,152]. However, diffusion-based imaging has its challenges and is not without limitations. In areas where fibers cross, branch, or feature complex anatomical configurations, tensor models often perform poorly, leading to inaccuracies in pathway reconstructions. These limitations have spurred the development of alternative techniques such as probabilistic diffusion tractography with multiple fiber orientations and spherical deconvolution [153]. Implementation of these methods, however, requires specialized hardware, extended scanning time, and intricate post-processing pipelines that exceed the capabilities of most clinical centers. Moreover, most diffusion sequences utilize echo-planar imaging, which can produce significant distortion artifacts. These technical constraints, combined with a low test-retest reliability likely explain the lack of prospective trials exploring the clinical utility of tractography [144]. Indeed, Jakab et al. showed that when using dMRI tractography to define the VIM in individual brains, the variance by use of different MRI scanners was higher than interindividual differences between subjects [154]. In our view, this critical study renders the prospect of using dMRI to identify individual anatomical differences of the CTT and Vim pessimistic with current technology. Hence, while dMRI offers invaluable insights into the structural connections within the brain and remains a cornerstone in neuroimaging research, its current limitations hinder patient-specific clinical adoption.

Beyond diffusion MRI, recent years have witnessed significant improvements in direct visualization of thalamic nuclei. Besides increases in magnetic field strength, this advancement can be attributed to optimized acquisition protocols and post-processing pipelines to enhance gray/white matter contrast and exploit differences in tissue composition, such as iron content (Fig. 8). Optimization of the inversion time allowing suppression of gray matter, in particular, has proven effective in delineating gray-white matter boundaries allowing the distinction of individual thalamic nuclei [155,156]. Recently, evidence has been accumulating that advanced imaging sequences can be exploited in clinically meaningful ways. Using fast gray matter acquisition T1 inversion recovery (FGATIR) sequences three independent groups identified and validated a hypointense region located at the base of Vim for surgical targeting during DBS surgery [102,157,158]. This hypointensity is currently thought to be the imaging equivalent of the CTT. Consistent with the description of the CTT, the hypointensity traverses PSA in anterolateral to posteromedial direction, adjoining the posterior borders of subthalamic nucleus and ZI rostrally, while being caudally confined by the ventral posteromedial and ventral posterior inferior thalamic nuclei [61,91] (Fig. 8). The hypointensity was reliably and consistently identified across patients and cohorts. More importantly, however, overlap of stimulation volumes with the identified hypointensity explained significant amounts of variance in observed clinical outcome following Vim DBS [102]. The predictive power of the hypointensity was greatest when accounting for inter-individual neuroanatomical variability in individual DBS patients, suggesting the ability of the imaging marker to account for anatomical differences. The latter was further emphasized in an anecdotal report by Middlebrooks et al. who reported improved tremor suppression following repositioning of a suboptimally placed DBS lead in Vim [157].

Advanced imaging sequences, such as FGATIR or white-matter nulled sequences, present potential solutions to some of the challenges associated with imaging fiber bundles in clinical settings. First, FGATIR allows high-resolution, isotropic, 3D slice-visualization of the Vim target at the single-millimeter level [155]. The best dMRI resolutions employed in clinical settings today are at the order of 1.5 ​mm at best. Second, the integration of FGATIR into the clinical workflow is straightforward, as acquisition does not require extensive postprocessing techniques or specialized equipment. It is compatible with any standard clinical picture archiving and communication system (PACS) or stereotactic planning software. Third, while the acquisition time of FGATIR is longer than T1w or T2w sequences, it remains more time-efficient than traditional dMRI protocols, which can demand up to nine times longer scanning durations [159]. Synthetic image generation may further improve workflows rendering considerations of scan time obsolete [160]. Lastly, tractography-based identification of CTT often produces major streamlines extending from the cerebellum to motor cortex, neglecting synaptic relay stations and crossing fibers. The absence of anatomical constraints consequently impedes the direct visualization of a precise surgical target during planning. Conversely, FGATIR and WMnull provides a circumscribed visual marker within PSA that is readily discernible and facilitates consistent targeting across all three planes.

## The therapeutic results of DBS of the region involving Vim/PSA

### The perspective of evidence-based medicine on DBS for ET

DBS for tremor has been increasingly applied since 1987. While numerous studies, both small and large, have been published (as highlighted in reviews [[161], [162], [163]]), controlled randomized trials comparing DBS to medical therapy are lacking. This absence is somewhat paradoxical but can be explained by the evaluation of DBS efficacy in prior comprehensive multi- or monocentric studies [90,[164], [165], [166], [167], [168]]. The endorsement of ET-DBS by both European regulatory authorities in 1993 and FDA in 1997 underscores its recognized therapeutic potential [81], although the FDA approved the treatment in 1997 based on two large non-randomized and uncontrolled multicenter trials. The important outcome was the blinded assessment of the stimulation-ON versus –OFF condition which showed coherent results (Fig. 9A and B) and the one year course of ON/OFF evaluations in a total of 167 patients with ET or PD [81].

**Fig. 10:**
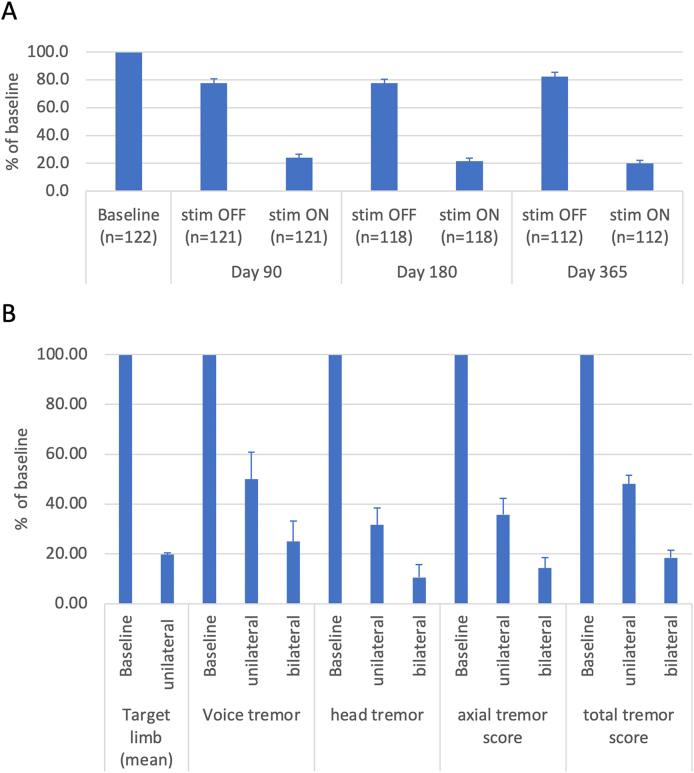
**Main results of the Abbott/St. Jude study. (A) Extend and persistence of the main effect on lateralized symptoms.** Unilateral stimulation in the whole study-cohort of 122 patients showed a profound effect on the lateralized tremor score which persisted over 90, 180 and 365 days [167]. The stim-OFF score is 21 ​% lower than the baseline score, which is most likely attributable to a microlesion effect induced during electrode placement intraoperatively. Notably for this study this main result was confirmed by a randomized blinded evaluation of the video-recorded tremor assessment of a subgroup (n ​= ​76) at baseline and 90 days [170]. **(B)** Bilateral stimulation has a stronger effect on axial symptoms than unilateral stimulation. The axial manifestations of essential tremor are captured at 6 months after first side and 3 months after second side surgery by the axial score and differ for uni- and bilateral stimulation for the subgroup of 38 patients with bilateral stimulation (All bars represent mean ​± ​SEM in percent of the respective baseline scores).

The evidence-based review of the Movement Disorder Society (MDS) provides a comprehensive summary of the current state of the art [169]. Initially, this review aimed to assess DBS for a range of clinical indications, including unilateral and bilateral lateralized symptoms (mainly hand and arm tremor) and axial tremor (predominantly leg tremor, balance, head, face, and voice tremor). However, due to limited data, the MDS team narrowed their focus on lateralized tremor symptoms. The conclusion drawn was that unilateral Vim-DBS is likely efficacious and potentially beneficial with an acceptable risk under specialized monitoring, representing the highest recommendation level for surgical procedures [169]. In contrast, bilateral Vim-DBS was deemed to have insufficient evidence, a striking discrepancy given that bilateral Vim implantation is more commonly performed in many centers internationally, as compared to unilateral DBS. Since the closure of the database for the EBM review in 2017, numerous smaller studies have emerged, but only one significant prospective non-randomized study is notable [167,170,171]. We chose to illustrate the main findings of the recent Abbot/St. Jude trial, because it is the largest, multicenter, uncontrolled prospective trial to date (Fig. 10). This trial reported a 84 ​% improvement for the lateralized items [167], in line with previous reports establishing DBS as an effective treatment for lateralized symptoms [161,162]. Importantly, this study also found significant improvements in quality of life and depression following unilateral Vim-DBS. A secondary analysis focusing on patients undergoing staged bilateral treatment revealed a 52 ​% improvement in total tremor scores six months post-unilateral stimulation and 82 ​% improvement post-bilateral stimulation relative to baseline [170].

Thus, these data confirm that unilateral DBS is effective, and bilateral DBS offers additional significant benefits.

In contrast to lateralized symptoms, axial symptoms, such as head or voice tremor, often do not respond as favourably to unilateral stimulation [161,163,171]. The recent study involving 38 patients undergoing staged lead implantation reported a 65 ​% improvement in axial tremor scores six months post-unilateral DBS and 85 ​% improvement post-bilateral stimulation as compared to baseline [171]. Specifically, head tremor improved by 68 ​% post-unilateral and 89 ​% post-bilateral stimulation, while voice tremor improved by 50 ​% and 75 ​%, respectively. These significant improvements in all relevant axial domains underscore the efficacy of bilateral DBS. It is worth noting that bilateral stimulation was associated with a higher incidence of adverse events, particularly during staged procedures, and that unilateral stimulation may be sufficient for some patients. Nonetheless, many centers opt for bilateral lead implantations from the outset. While overall improvements vary across studies, the literature supports the concept that bilateral stimulations yield superior outcomes.

We note that centers have demonstrated considerable variability in the choice of target, and reported outcomes often reflect combined data for Vim and PSA implantations (Table 3). Studies controlling for this distinction are reported separately.

### How might thalamic and PSA-interventions affect tremor?

The mechanisms underlying DBS for tremor suppression are intricate and remain largely elusive, but they are undoubtedly intertwined with the pathophysiology of ET. Several hypotheses have been proposed in association with ET pathophysiology including neurodegeneration and neurotransmitter imbalances [130]. The neurodegeneration hypothesis suggests a possible cerebellar disease component, evidenced by loss of Purkinje cells and accumulation of Lewy bodies in some ET patients [172]. However, these findings are not universally accepted, and the debate continues regarding ET's classification as a neurodegenerative disorder. Separately, both GABAergic and glutamatergic dysfunctions have been implicated in ET pathogenesis. The GABA hypothesis posits that GABAergic dysfunction, particularly in the cerebellar dentate nucleus, significantly influences ET. This theory is supported by the effectiveness of GABA-enhancing drugs [162], reduced GABA levels in cerebrospinal fluid [173], and altered GABA receptor binding in neuroimaging studies [174]. Reduced GABA receptor activity is proposed to lead to overactivity in deep cerebellar nuclei and the cerebellothalamocortical circuit, culminating in tremor. Synaptic pruning deficits between climbing fibers and Purkinje cells resulting from glutamate receptor (GluRδ2) insufficiency is an alternate hypothesis [175]. Reduced GluRδ2 expression may lead to excessive cerebellar oscillations, contributing significantly to ET tremor generation.

In addition to molecular mechanisms, system-oriented research proposed pathological oscillatory network activity in ET. Based on pioneering work by Llinas and colleagues who uncovered the autonomous rhythmicity of cells within the inferior olive [176,177], studies investigating pathological oscillatory activity in ET have initially focused on this structure. However, numerous subsequent studies have contested this initial perspective, challenging the notion that (pathological) tremor is solely driven by a single oscillator [130,178]. This shift in understanding has redirected attention towards network properties and has been summarized in the *oscillating network hypothesis*. This hypothesis posits that tremor arises from interactions within the cerebellothalamocortical circuit, with components dynamically entraining each other [129,130,179]. The most convincing evidence in favour of such circular excitation comes from microelectrode recordings within the thalamus and the basal ganglia in patients suffering from various forms of tremor [75,180,181]. A synthesis of electrophysiological, functional, and structural imaging studies has led to the proposition of a ‘tremor loop’ for action tremors. This loop interconnects cortex, cerebellum, and thalamus, with the cerebellothalamic pathway emerging as one critical link in this circuit. The occurrence of disease-related lesions within this loop has been associated with the cessation of tremor, as evidenced by the impact of small, strategic strokes [182]. The success of stimulation and therapeutic lesions further reinforces this conceptual framework [183].

Recently, Milosevic et al. provided mechanistic insights into the local effects of Vim-DBS. Specifically, the authors implanted a pair of closely spaced microelectrodes into Vim to simultaneously record tremor-synchronous activity of thalamic cells and perform intermittent electrical stimulation [74]. Stimulation at 100 ​Hz initially drove neural activity, but soon interrupted tremor-synchronous activity, leading to a suppression of peripheral tremor. The observed response aligns with the concept of synaptic fatigue due to the overstimulation of input to Vim-neurons, providing a coherent explanation for the profound efficacy of DBS at the local level. Alternatively, lesioning the afferent fibers offers another avenue to neutralize the pathological cerebello-thalamic input. The precise downstream effects resulting from synaptic depletion as well as effects induced by antero- and retrograde entrainment remain unclear, however, and are subject to future research.

Above and beyond models on the cell level, functional imaging studies have cumulated in what is now referred to as the dimmer-switch model, which states that cerebellar input to the thalamus codes for the occurrence of tremor while basal ganglia input for tremor amplitude [131].

### Adverse events and complications of Vim-DBS

Electrophysiological recordings and advanced imaging modalities have significantly improved the precision and efficacy of thalamic targeting in ET. However, challenges persist, particularly in preventing side-effects associated with Vim-DBS. Such adverse effects narrow the therapeutic window and, in severe cases, render symptom control unattainable. Electrical stimulation of Vim and PSA have been linked with several adverse side-effects, including speech impairment, disequilibrium, sensory deficits such as paresthesia and numbness, weakness, and dysgeusia [184]. While many of these complications are reversible by means of reprogramming, persistence has been reported in up to 14 ​% of patients, despite the employment of diverse programming strategies.

One of the most prevalent side-effect reported during Vim-DBS is dysarthria, which occurs in up to 39 ​% of patients with unilateral implantation [185,186] and 75 ​% of patients undergoing bilateral DBS [[187], [188], [189]]. Dysphasia and hypophonia are less common and predominantly occur during bilateral stimulation (dysphasia: 10 ​% unilateral, 19 ​% bilateral; hypophonia: 5 ​% unilateral, 19 ​% bilateral) [186]. Despite a wealth of clinical data and experience, the anatomical substrates of this side-effect remain incompletely understood. Current hypotheses have attributed their origin to the spread of current to the internal capsule laterally, interference with the CTT inferiorly, and medial thalamic stimulation with current encroaching onto the face, mouth, and tongue representation as well as pallidothalamic fibers ascending into medial aspects of the motor thalamus [[190], [191], [192], [193], [194]].

Ataxia, particularly gait ataxia, is another frequently observed side-effect of Vim-DBS, with a reported prevalence of 56%–86 ​% in the literature [187,188]. Intriguingly, while gait ataxia is exacerbated under supratherapeutic stimulation conditions (i.e., increased amplitude and pulse width), it has not been shown to deteriorate under standard therapeutic stimulation regimens when compared to the stimulation-OFF state [195]. This phenomenon of overstimulation has been linked to excessive entrainment of the cerebello-thalamo-cortical loop, especially during ventral thalamic DBS.

Sensory disturbances such as paresthesia have been documented in up to 45 ​% of ET patients. They typically arise from stimulation of the sensory thalamic nuclei, namely VPL, leading to localized symptoms in regions such as the face, arm, or leg [187,196]. Moreover, paresthesia has been associated with current encroachment onto the medial lemniscus inferiorly, which typically manifest in the form of hemibody symptoms. Other less commonly reported side-effects of Vim-DBS include hemiparesis [184] (unilateral: 5 ​%, bilateral 7 ​%), decline in verbal fluency [197], and dysgeusia [198]. The latter has been associated with posterior stimulation, potentially extending into VPL or VPM, as well as the involvement of the trigemino-thalamic tract and medial lemniscus, which are believed to relay – among other information – gustatory sensation. De Vloo et al. specifically implicated the solitariothalamic gustatory fibers within medial lemniscus to be causally involved in dysgeusia [199]. In contrast to Vim proper, the side-effects induced by stimulation are typically experienced at lower stimulation intensities during PSA-DBS, but have a similar incidence and quality [97,200,201].

To mitigate stimulation-induced side-effects in the context of ET-DBS, the implementation of directional leads has been a significant advancement. These leads enable directional stimulation, offering a potential solution to correct misplacements of up to 1 ​mm [202]. Preliminary evidence indicates that directional stimulation broadens the therapeutic window and reduces the necessary current intensity as compared to omnidirectional stimulation, by focusing stimulation to the target region and reducing current spread to adjacent eloquent structures.

Surgery-related complications of ET-DBS are consistent with lead implantations in other locations [203], though they are likely less frequent for ET than for PD [204]. Elble et al. provided a comprehensive summary of complications in 661 patients from a US database of operations occurring between 2000 and 2009 [161]. 7.1 ​% of patients experienced at least one complication, including wound infection (3.0 ​%), pneumonia (2.4 ​%), haemorrhage (1.5 ​%), pulmonary embolism (0.6 ​%), lead replacement or revision (0.3 ​%), and generator removal or revision (1.1 ​%). One patient (0.2 ​%) passed away within 90 days post-operation. Notably, there was no significant correlation between these complications and patient age, which ranged from under 50 to 90 years, although only a fraction (5.6 ​%) of patients were between 80 and 90 years old.

### Loss of stimulation benefit and habituation

The energy required for effective stimulation in ET, as well as the number of active contacts, often show gradual increases over time [205,206]. Notably, the rate of this increment is more pronounced in ET compared to other tremors, such as those associated with PD or post-stroke tremors [207]. This differential response may be indicative of a long-term benefit decay uniquely manifesting in ET-DBS patients. Indeed, diminishing efficacy during chronic DBS appears to be a common phenomenon in Vim-DBS, which has been ascribed to various factors, including DBS tolerance, disease progression, aging, and brain atrophy. The concept of habituation or tolerance to DBS in particular has been acknowledged since the inception of Vim-DBS [208], however, its precise pathophysiological underpinnings remain elusive. Possible disease-related mechanisms include tremor etiology and its inherent prognosis, while surgical factors such as electrode placement, microlesion effect, and placebo effect may also play a role [209].

A limited number of long-term studies were able to confirm a sustained long-term reduction of tremor in most patients following DBS, although a decline from initial improvement levels was noted in a subset of patients [188,206]. In contrast, a larger body of literature points towards waning benefit during chronic DBS. Indeed, data from the longest follow-up study investigating stimulation benefit over a period of 18 years revealed a decreased benefit from 66 ​% in the first year to 48 ​% at the final follow-up [210]. Several other studies have confirmed this notion: In a cohort of 45 patients, Shih et al. reported a diminished benefit in 73 ​% of Vim-DBS recipients by telephone interview at some point during a 5-year follow-up [205]. A detailed evaluation of Vim-DBS effects on activities of daily living in 19 ​ET patients revealed that initial improvements observed at the one-year benchmark were largely negated in subsequent years, with the sole exception being the ability to eat, which remained improved even after seven years [211]. Paschen et al. confirmed these findings noting a tremor reduction from 50 ​% at three years to a mere 15 ​% a decade post-DBS implantation in a cohort of 20 patients [212]. Of note, long-term worsening of symptoms was more profound during the stim-ON phase compared to the stim-OFF condition in this study, hinting at a potential habituation to stimulation. Indeed, more dramatic declines in stimulation benefit due to habituation have been noted in subsets of patients [205], which occurred as early as ten weeks following stimulation initiation [213]. The term ‘escapers’ has recently been proposed to characterize the phenomenon of diminished response to stimulation following an initial period of effective tremor suppression, both in early and late stages of DBS [206].

Several strategies have been suggested to prevent the occurrence of tolerance, including the nightly deactivation of the DBS device [207,214]. However, empirical evidence supporting this intervention remains sparse. Some investigators have advocated for on-demand DBS as a means to mitigate or manage habituation [215,216], while other groups have proposed alternating between different yet equally effective stimulation paradigms [217,218]. Reprogramming (e.g., reduction of the pulse with <60 μs) may be efficacious when escalation of DBS is constrained by stimulation-induced side effects, using new devices that facilitate a more elaborate shaping of stimulation volumes. Finally, a study involving seven patients demonstrated that stimulation of Voa in ET patients, who had developed habituation to Vim stimulation, yielded an additional tremor reduction of 16.7 ​% compared to Vim stimulation alone [219]. To achieve sufficient spatial coverage, however, the authors relied on dual thalamic lead implantation, a strategy not universally endorsed for ET treatment.

The challenge of habituation and loss of stimulation benefit remains a significant hurdle in chronic treatment of ET. For PD, such habituation has not been described in STN and GPi-DBS. So far it is unknown if such habituation occurs more often and/or profound during Vim-proper or PSA stimulation. It is important to note, however, that even if long-term habituation occurs, there is still a major significant improvement between the stim-ON vs stim-OFF state. As the field continues to evolve, finding reliable solutions to this issue will be paramount to optimizing long-term patient outcomes.

In summary, Deep Brain Stimulation (DBS) has proven to be an effective intervention for Essential Tremor (ET), with a growing body of literature suggesting that the optimal target for tremor control is in the PSA and not the classic target in the Vim. The cerebello-thalamo-cortical loop appears to be causally involved in tremor pathophysiology. Electrophysiological techniques and advanced imaging sequences have drastically improved our ability to identify the anatomical target, however, uncertainty remains regarding which exact anatomical substrates (i.e., tracts and nuclei) are involved in tremor control. The variable subdivisions and terminology introduced for the motor thalamus have hampered our understanding of stimulation results. The newly developing field of MRgFUS treatment for tremor [220] is targeting the very same region of the thalamus and PSA. But, to date, it is not clear whether lesioning the PSA or the Vim proper is more efficient. Critically, the approach differs from both DBS and radiofrequency lesioning as suggested by a first pathoanatomic study [221].

There is a matter of nomenclature whose resolution would benefit the field. Hassler and colleagues used the term subthalamus for the PSA/zona incerta and the broader region below the thalamus excluding the subthalamic and red nucleus. Recent approaches of lesioning the subthalamic nucleus using focused ultrasound surgery, however, have now employed the term ‘subthalamotomy’, which introduces potentially confusing nomenclature when considering the historical trajectory [53]. While the established use of pallidotomy and thalamotomy supports using the term subthalamotomy as a label for the lesion of the subthalamic nucleus, we encourage other investigators to explicitly define the intended target for lesioning in future reports, in recognition that much of the subthalamic literature related to ET treatment is focused on the region rather than the nucleus.

Important challenges that remain to be addressed include loss of benefit to DBS and habituation. Clear treatment strategies for these cases are needed. Concepts that could be helpful in this regard include refined DBS techniques, closed-loop paradigms, and a deeper understanding of ET pathophysiology.

### Author contributions

Conceptualization: CN, AH, GD; Writing-Original Draft: CN, AH, GD; Writing-Review and Editing: CN, KIK, II, SP, AKH, GRC, RMR, AH, GD; Visualization and Illustration: CN, AH, KIK, II, GD; Supervision: AH, GD.

## Declaration of competing interest

The authors declare the following financial interests/personal relationships which may be considered as potential competing interests: Guenther Deuschl reports a relationship with Boston Scientific Corp that includes: consulting or advisory. Guenther Deuschl reports a relationship with Cavion that includes: consulting or advisory. Guenther Deuschl reports a relationship with Functional Neuromodulation that includes: consulting or advisory. Guenther Deuschl reports a relationship with Thieme Medical Publishers that includes: consulting or advisory. Andreas Horn reports a relationship with German Research Foundation that includes: funding grants. Andreas Horn reports a relationship with Deutsches Zentrum für Luft-und Raumfahrt that includes: funding grants.
